# A cybercatalogue of American sand fly types (Diptera, Psychodidae, Phlebotominae) deposited at the Natural History Museum, London

**DOI:** 10.3897/BDJ.6.e24484

**Published:** 2018-05-15

**Authors:** Zoe J. O. Adams, Paloma Helena Fernandes Shimabukuro

**Affiliations:** 1 The Natural History Museum, Department of Life Sciences, South Kensington, SW7 5BD, United Kingdom; 2 Grupo de Estudos em Leishmanioses/Coleção de Flebotomíneos - FIOCRUZ/COLFLEB, Belo Horizonte, MG 30190-002, Brazil

**Keywords:** Taxonomy, Sand fly, Neotropical, Nearctic, Phlebotominae, type catalogue, NHMUK

## Abstract

**Background:**

Sand flies (Diptera, Psychodidae, Phlebotominae) are biting flies involved in the transmission of pathogens, including the protozoan parasite *Leishmania* amongst human and non-human animals (Rangel and Lainson 2009).

**New information:**

A total of 60 species of American Phlebotominae (Diptera: Psychodidae), distributed amongst 16 genera were studied. A checklist of the primary and secondary type specimens held at the Natural History Museum, London (NHMUK), is given and 968 photographs of the specimens and their labels are made available on a Scratchpads website http://phlebotominaenhmtypes.myspecies.info.

## Introduction

There are approximately 1,000 valid described species of sand flies in the world, of which 530 are known to occur in the Americas (Shimabukuro et al. 2017).

The aim of this work is to provide a checklist of Phlebotominae types of the Neotropical and Nearctic regions held in the collections of the Natural History Museum, London (NHMUK formerly BMNH), together with electronic links to, scans of whole slides, plus detail images of the taxonomically informative morphological structures. This work updates the American portion of the 1990 checklist of types deposited at the NHMUK (Townsend and Lane 1990).

### History

Owing to the small size and morphological homogeneity of phlebotomine sand flies, few species were described before the turn of the 20^th^ century. Indeed little progress in their taxonomy was made until Adler and Theodor (1926) and Theodor (1932) drew attention to the utility of various internal structures (cibarium & spermathecae) for species differentiation. Currently, taxonomically valid species descriptions of phlebotomine sand flies still rely on adult morphology. Although newer molecular techniques are increasingly used for identifying species (Zapata et al. 2012), they have yet to be formally used as characters in species descriptions. Type specimens are confined to the adult stage, with few taxa described in the immature stages.

Significant donations to the NHMUK collection have come from: (i) Graham Bell Fairchild who worked on the Panamanian fauna in the 1950s and 60s; (ii) a number of early types described by Robert Newstead in the 1920s when he was a Professor at the Liverpool School of Tropical Medicine; (iii) and most significantly, the collection of Oskar Theodor, which the museum purchased in 1981 after his retirement from the University of Jerusalem. The NHMUK has also absorbed the specimen collections of the London School of Tropical Medicine and Hygiene (LSHTM).

**Figure 1.**
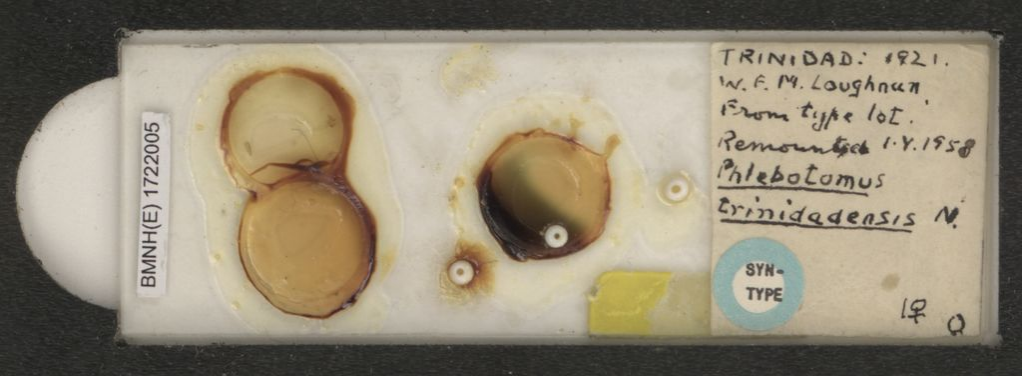
Example of an R. Newstead type remounted into Berlese by D. J. Lewis, showing the typical darkening of the mountant as it deteriorates.

The main body of the NHMUK Phlebotominae collection was assembled by Dr D.J. Lewis who, though not a member of staff, was based at the museum for 30 years (1956-1986) whilst in the employ of the Medical Research Council in London. All his material was mounted in Berlese media which is not a permanent mountant. There are many examples of deteriorating Lewis slides in the NHMUK collection (Fig. 1), as well as instances where Lewis remounted other authors' type specimens into Berlese. His distinctive handwriting and messy slides, often bearing multiple specimens, are instantly recognisable.

### Taxonomic treatment

Phlebotominae taxonomy has a long history of differences in opinion amongst experts and various conflicting classification schemes. The current situation is no exception, with both classification of Young and Duncan (1994), which is based on the previous work of Lewis et al. (1977) and that of Galati (2003b) in use at the same time, for the New World fauna. For a comparison of how these two classifications relate to each other see Fig. 2.

Currently, the NHMUK collections are arranged according to Young and Duncan (1994), but we have used the Galati (2003b) naming systems here to reflect the current knowledge of the group.

In their guide to identification and distribution, Young and Duncan (1994) adopt the classification of Lewis et al. (1977) with only minor changes. They make it clear that their arrangement of taxa does not represent a phylogenetic analysis of the group and, aside from a few comments in the notes, give no information on the relationships between their subgeneric groups. It is clear that their groupings have been arrived at through a phenetic review of morphological characters with little focus on identifying any common ancestry between groups.

Lewis et al. (1977) proposed their classification at a time when those working on sand flies were faced with the choice between two family names, several subfamily names and, for some species, three possible generic names. Their aim was to create a stable general framework with flexible subdivisions into which, recent and future ideas on the evolutionary relationships within the group, could be assimilated. They present a review of the early literature on the group and, with reference to the International Code of Zoological Nomenclature (ICZN), proposed the use of the family name Psychodidae Newman 1834 and the subfamily name Phlebotominae Rondani 1840, proposals that have been more or less universally accepted. Their proposal for the use of just five genera (*Brumptomyia*, *Lutzomyia*, *Phlebotomus*, *Sergentomyia* and *Warileya*) now seems less successful.

At that time, 1977, around 600 sand fly species had been described, with 290 of these in the American genus *Lutzomyia* (Young and Duncan 1994). The current checklist for American sand flies alone (Shimabukuro et al. 2017) stands at 530 and use of the Lewis et al. (1977) concept of the genus *Lutzomyia* would place 495 of these taxa into a single genus. It now seems likely that, by placing the emphasis on a stable and simple classification over one that best represented the evolutionary history of the group, Lewis et al. (1977) created a number of polyphyletic groups, particularly their genus *Lutzomyia*.

The classification of Galati (1995) and Galati (2003a) is more complex with the genus *Lutzomyia*, as defined in Lewis et al. (1977) and Young and Duncan (1994), split into 23 genera and 22 sub-generic groupings and has been increasingly utilised by authors working on the American fauna.

The study by Galati (1995) represented the first comprehensive attempt to perform a phylogenetic analysis of the American Phlebotominae, comprising a cladistic analysis of 88 adult morphology characters, using the Psychodidae subfamily Bruchomyiinae as the principal outgroup. The classification has since been updated and represents our current best understanding of the evolutionary relationships within this important group (Galati 2003a, Galati 2003b).

There are some nomenclatural issues with the Galati classification as it stands at the present moment (see Shimabukuro et al. 2017). Within the classification a number of family group and genus group names are attributed to Artemiev from his 1991 work (Artemiev 1991). In this work, Artemiev does not accompany any of his new names with a description that states in words characters that are purported to differentiate the taxa, thus all his new names do not comply with ICZN Article 13.1.1 http://www.iczn.org/iczn/index.jsp and should be considered nomina nuda. Likewise, there are a large number of family group and genus group names in the classification attributed to Galati (1995), where she also fails to accompany any of her new names with a description that can be used to distinguish them. Galati lists the characters used, gives a polarised transformation series and maps the characters and taxa on to a cladogram, but this is not sufficient under the code to render the new names from her 1995 work available. In 2003, Galati updated her 1995 classification (Galati 2003b, Galati 2003a), this 2003 work contains extensive identification keys for all groups and any unavailable names should take authorship and date from this later act of establishment under ICZN articles 50 and 21.

## Materials and methods

### Checklist

The list contains information compiled from the examination of Nearctic and Neotropical species of sand flies from the Diptera Collection at the NHMUK.

Species are listed alphabetically, with genus and subgenus designations according to Shimabukuro et al. 2017. Links to the scratchpad entry and images for each specimen are given in the **eventRemarks** section of the checklist.

Readers familiar with the classification of Young and Duncan (1994) should refer to the scratchpads website http://phlebotominaenhmtypes.myspecies.info where the specimens have been arranged according to both classifications.

We have excluded from this catalogue two NHMUK specimens bearing “type” labels, for which we can find no record of the given species name in the literature. One labelled as the holotype of *Lutzomyia witremundoi* (author Feliciangeli), the other labelled as a paratype of *L. patiñoi* (authors Mangabeira & Galindo). It seems probable that these are unpublished manuscript names and are therefore unavailable under article 8 of the ICZN.

### Specimen Images

Photographs were taken in the Sackler Biodiversity Imaging Laboratory (SBIL), at the NHMUK, with an Olympus BX63-CBH microscope equipped with a DP73 camera and differential interference contrast (DIC) illumination. The images were edited in the Cellsens software and stacked images were produced by HeliconFocus 6 software.

Whole slide scans were generated using a SatScan instrument at x5 magnification and images were cropped and processed using the Inselect software, version 0.1.35, available from the Natural History Museum https://naturalhistorymuseum.github.io/inselect.

## Data resources

### Bibliography

Most of the original species descriptions are still under copyright to their original publisher. The bibliographical and historical database of scientific articles on leishmaniases and sand flies hosted by the Laboratoire de Parasitologie at the University of Montpellier is an extremely useful resource http://www.leishpub.univ-montp1.fr.

### Specimen Images

The specimen images are available on the NHMUK data portal: https://doi.org/10.5519/0051226.

The checklist, specimen data and specimen images are available on a Scratchpads website http://phlebotominaenhmtypes.myspecies.info.

## Checklists

### Checklist

#### Pressatia acanthobasis

Fairchild & Hertig, 1952

Phlebotomus acanthobasis Fairchild & Hertig, 1952 (Fairchild and Hertig 1952)

##### Materials

- **Type status:** Paratype. **Occurrence:** catalogNumber: BMNHE1722073; sex: Female; **Taxon:** scientificName: *Pressatia acanthobasis* (Fairchild & Hertig, 1952); acceptedNameUsage: *Pressatia camposi* (Rodríguez, 1950); **Location:** country: Panama; stateProvince: Colón; locality: Mojinga swamp; **Event:** eventDate: 09-05-51; eventRemarks: http://phlebotominaenhmtypes.myspecies.info/node/192; **Record Level:** institutionCode: NHMUK; basisOfRecord: Preserved Specimen
- **Type status:** Paratype. **Occurrence:** catalogNumber: BMNHE1722074; sex: Male; **Taxon:** scientificName: *Pressatia acanthobasis* (Fairchild & Hertig, 1952); acceptedNameUsage: *Pressatia camposi* (Rodríguez, 1950); **Location:** country: Panama; stateProvince: Panamá; locality: Rio Majé, Charco el Toro; **Event:** eventDate: 03-22-50; eventRemarks: http://phlebotominaenhmtypes.myspecies.info/node/193; **Record Level:** institutionCode: NHMUK; basisOfRecord: Preserved Specimen

##### Distribution

Colombia, Costa Rica, Ecuador, Nicaragua, Panama

##### Notes

Synonym of *Pressatia camposi* (Rodríguez, 1950) Rodríguez 1950. Synonymised by Fairchild and Hertig 1958b.

#### Psathyromyia aclydifera

Fairchild & Hertig, 1952

Phlebotomus aclydiferus Fairchild & Hertig, 1952 (Fairchild and Hertig 1952)

##### Materials

- **Type status:** Paratype. **Occurrence:** catalogNumber: BMNHE1721992; sex: Female; **Taxon:** scientificName: Psathyromyia (Xiphopsathyromyia) aclydifera (Fairchild & Hertig, 1952); **Location:** country: Panama; stateProvince: Colón; locality: Mojinga swamp, nr. Gatún; **Event:** eventDate: 08-21-51; eventRemarks: http://phlebotominaenhmtypes.myspecies.info/node/111; **Record Level:** institutionCode: NHMUK; basisOfRecord: Preserved Specimen
- **Type status:** Paratype. **Occurrence:** catalogNumber: BMNHE1721993; sex: Male; **Taxon:** scientificName: Psathyromyia (Xiphopsathyromyia) aclydifera (Fairchild & Hertig, 1952); **Location:** country: Panama; stateProvince: Chiriquí; municipality: Barú; locality: Puerto Armuelles; **Event:** eventDate: 06-09-51; eventRemarks: http://phlebotominaenhmtypes.myspecies.info/node/112; **Record Level:** institutionCode: NHMUK; basisOfRecord: Preserved Specimen

##### Distribution

Belize, Bolivia, Colombia, Costa Rica, Ecuador, Guatemala, Honduras, Mexico, Nicaragua, Panama

##### Notes

Valid species in Psathyromyia (Xiphopsathyromyia).

#### Nyssomyia anduzei

Rozeboom, 1942

Phlebotomus anduzei Rozeboom, 1942 (Rozeboom 1942)

##### Materials

- **Type status:** Syntype. **Occurrence:** catalogNumber: BMNHE1722068; sex: Female; **Taxon:** scientificName: *Nyssomyia anduzei* (Rozeboom, 1942); **Location:** country: Venezuela; stateProvince: Bolívar; municipality: Gran Sabana; locality: Gran Sabana; **Event:** year: 1941; eventRemarks: http://phlebotominaenhmtypes.myspecies.info/node/187; **Record Level:** institutionCode: NHMUK; basisOfRecord: Preserved Specimen
- **Type status:** Syntype. **Occurrence:** catalogNumber: BMNHE1722120; **Taxon:** scientificName: *Nyssomyia anduzei* (Rozeboom, 1942); **Location:** country: Venezuela; stateProvince: Bolívar; municipality: Gran Sobana; locality: Gran Sobana; **Event:** year: 1941; month: 12; eventRemarks: http://phlebotominaenhmtypes.myspecies.info/node/239; **Record Level:** institutionCode: NHMUK; basisOfRecord: Preserved Specimen
- **Type status:** Syntype. **Occurrence:** catalogNumber: BMNHE1722121; sex: Female; **Taxon:** scientificName: *Nyssomyia anduzei* (Rozeboom, 1942); **Location:** country: Venezuela; stateProvince: Bolívar; municipality: Gran Sobana; locality: Gran Sobana; **Event:** year: 1941; month: 12; eventRemarks: http://phlebotominaenhmtypes.myspecies.info/node/240; **Record Level:** institutionCode: NHMUK; basisOfRecord: Preserved Specimen
- **Type status:** Syntype. **Occurrence:** catalogNumber: BMNHE1722122; sex: Female; **Taxon:** scientificName: *Nyssomyia anduzei* (Rozeboom, 1942); **Location:** country: Venezuela; stateProvince: Bolívar; municipality: Gran Sobana; locality: Gran Sobana; **Event:** year: 1941; month: 12; eventRemarks: http://phlebotominaenhmtypes.myspecies.info/node/241; **Record Level:** institutionCode: NHMUK; basisOfRecord: Preserved Specimen
- **Type status:** Syntype. **Occurrence:** catalogNumber: BMNHE1722123; **Taxon:** scientificName: *Nyssomyia anduzei* (Rozeboom, 1942); **Location:** country: Venezuela; stateProvince: Bolívar; municipality: Gran Sobana; locality: Gran Sobana; **Event:** year: 1941; month: 12; eventRemarks: http://phlebotominaenhmtypes.myspecies.info/node/242; **Record Level:** institutionCode: NHMUK; basisOfRecord: Preserved Specimen

##### Distribution

Brazil, Costa Rica, French Guiana, Panama, Peru, Venezuela

##### Notes

Valid species in *Nyssomyia*.

#### Pintomyia aulari

Feliciangeli, Ordoñez & Manzanilla, 1984

Lutzomyia aulari Feliciangeli, Ordoñez & Manzanilla, 1984 (Feliciangeli et al. 1984b)

##### Materials

- **Type status:** Holotype. **Occurrence:** catalogNumber: BMNHE1722088; sex: Male; **Taxon:** scientificName: Pintomyia (Pifanomyia) aulari (Feliciangeli, Ordoñez & Manzanilla, 1984); **Location:** country: Venezuela; stateProvince: Trujillo; locality: Loma Abajo; **Event:** eventDate: 06-01-70; eventRemarks: http://phlebotominaenhmtypes.myspecies.info/node/207; **Record Level:** institutionCode: NHMUK; basisOfRecord: Preserved Specimen

##### Distribution

Venezuela

##### Notes

Valid species in Pintomyia (Pifanomyia).

#### Trichophoromyia auraensis

Mangabeira, 1942

Flebotomus auraensis Mangabeira, 1942 (Mangabeira 1942)

##### Materials

- **Type status:** Paratype. **Occurrence:** catalogNumber: BMNHE1722081; sex: Male; **Taxon:** scientificName: *Trichophoromyia auraensis* (Mangabeira, 1942); **Location:** country: Brazil; stateProvince: Pará; municipality: Belém; locality: Aurá, em buraco de tatu.; **Event:** year: 1940; month: 8; eventRemarks: http://phlebotominaenhmtypes.myspecies.info/node/200; **Record Level:** institutionCode: NHMUK; basisOfRecord: Preserved Specimen

##### Distribution

Bolivia, Brazil, Colombia, Peru, Surinam, Venezuela

##### Notes

Valid species in *Trichophoromyia*.

#### Psychodopygus bispinosus

Fairchild & Hertig, 1951

Phlebotomus bispinosus Fairchild & Hertig, 1951 (Fairchild and Hertig 1951a)

##### Materials

- **Type status:** Paratype. **Occurrence:** catalogNumber: BMNHE1251325; sex: Female; **Taxon:** scientificName: *Psychodopygus bispinosus* (Fairchild & Hertig, 1951); **Location:** country: Panama; stateProvince: Chiriquí; locality: La Victoria, Cerro Jefe; **Event:** eventDate: 08-29-50; eventRemarks: http://phlebotominaenhmtypes.myspecies.info/node/97; **Record Level:** institutionCode: NHMUK; basisOfRecord: Preserved Specimen
- **Type status:** Allotype. **Occurrence:** catalogNumber: BMNHE1251326; sex: Male; **Taxon:** scientificName: *Psychodopygus bispinosus* (Fairchild & Hertig, 1951); **Location:** country: Panama; stateProvince: Chiriquí; locality: La Victoria, Cerro Jefe; **Event:** eventDate: 08-30-50; eventRemarks: http://phlebotominaenhmtypes.myspecies.info/node/98; **Record Level:** institutionCode: NHMUK; basisOfRecord: Preserved Specimen

##### Distribution

Belize, Brazil, Colombia, Costa Rica, Ecuador, French Guiana, Guatemala, Honduras, Mexico, Nicaragua, Panama, Surinam

##### Notes

Valid species in *Psychodopygus*.

#### Lutzomyia botella

Fairchild & Hertig, 1961

Phlebotomus botellus Fairchild & Hertig, 1961 (Fairchild and Hertig 1961)

##### Materials

- **Type status:** Paratype. **Occurrence:** catalogNumber: BMNHE1722010; sex: Female; **Taxon:** scientificName: Lutzomyia (Helcocyrtomyia) botella (Fairchild & Hertig, 1961); **Location:** country: Panama; stateProvince: Chiriquí; locality: El Volcan; **Event:** eventDate: 03-23-54; eventRemarks: http://phlebotominaenhmtypes.myspecies.info/node/129; **Record Level:** institutionCode: NHMUK; basisOfRecord: Preserved Specimen

##### Distribution

Panama

##### Notes

Valid species in Lutzomyia (Helcocyrtomyia).

#### Brumptomyia brumpti

Larrousse, 1920

Phlebotomus brumpti Larrousse, 1920 (Larrousse 1920)

##### Materials

- **Type status:** Syntype. **Occurrence:** catalogNumber: BMNHE1722011; sex: Female; **Taxon:** scientificName: *Brumptomyia brumpti* (Larrousse, 1920); **Location:** country: Brazil; locality: Albuquerque; **Event:** eventRemarks: http://phlebotominaenhmtypes.myspecies.info/node/130; **Record Level:** institutionCode: NHMUK; basisOfRecord: Preserved Specimen
- **Type status:** Syntype. **Occurrence:** catalogNumber: BMNHE1722043; sex: Male; **Taxon:** scientificName: *Brumptomyia brumpti* (Larrousse, 1920); **Location:** country: Brazil; locality: Albaquerque; **Event:** eventRemarks: http://phlebotominaenhmtypes.myspecies.info/node/162; **Record Level:** institutionCode: NHMUK; basisOfRecord: Preserved Specimen

##### Distribution

Argentina, Bolivia, Brazil

##### Notes

Valid species in *Brumptomyia*.

#### Micropygomyia cayennensis braci

Lewis, 1967

Lutzomyia cayennensis braci Lewis, 1967 (Lewis 1967)

##### Materials

- **Type status:** Holotype. **Occurrence:** catalogNumber: BMNHE1722028; sex: Male; **Taxon:** scientificName: Micropygomyia (Micropygomyia) cayennensis braci (Lewis, 1967); **Location:** country: Cayman Islands; stateProvince: Cayman Brac; locality: Bamboo Bay; **Event:** eventDate: 12-18-64; eventRemarks: http://phlebotominaenhmtypes.myspecies.info/node/147; **Record Level:** institutionCode: NHMUK; basisOfRecord: Preserved Specimen
- **Type status:** Paratype. **Occurrence:** catalogNumber: BMNHE1722050; sex: Female; **Taxon:** scientificName: Micropygomyia (Micropygomyia) cayennensis braci (Lewis, 1967); **Location:** country: Cayman Islands; stateProvince: Cayman Brac; locality: Bamboo Bay; **Event:** eventDate: 18/12/64; eventRemarks: http://phlebotominaenhmtypes.myspecies.info/node/169; **Record Level:** institutionCode: NHMUK; basisOfRecord: Preserved Specimen
- **Type status:** Paratype. **Occurrence:** catalogNumber: BMNHE1722051; sex: Female; **Taxon:** scientificName: Micropygomyia (Micropygomyia) cayennensis braci (Lewis, 1967); **Location:** country: Cayman Islands; stateProvince: Cayman Brac; locality: Bamboo Bay; **Event:** eventDate: 18/12/64; eventRemarks: http://phlebotominaenhmtypes.myspecies.info/node/170; **Record Level:** institutionCode: NHMUK; basisOfRecord: Preserved Specimen
- **Type status:** Paratype. **Occurrence:** catalogNumber: BMNHE1722106; sex: Female; **Taxon:** scientificName: Micropygomyia (Micropygomyia) cayennensis braci (Lewis, 1967); **Location:** country: Cayman Islands; stateProvince: Cayman Brac; locality: Stake Bay; **Event:** eventDate: 12-16-64; eventRemarks: http://phlebotominaenhmtypes.myspecies.info/node/225; **Record Level:** institutionCode: NHMUK; basisOfRecord: Preserved Specimen
- **Type status:** Paratype. **Occurrence:** catalogNumber: BMNHE1722107; sex: Male; **Taxon:** scientificName: Micropygomyia (Micropygomyia) cayennensis braci (Lewis, 1967); **Location:** country: Cayman Islands; stateProvince: Cayman Brac; locality: Bamboo Bay; **Event:** eventDate: 12-18-64; eventRemarks: http://phlebotominaenhmtypes.myspecies.info/node/226; **Record Level:** institutionCode: NHMUK; basisOfRecord: Preserved Specimen
- **Type status:** Paratype. **Occurrence:** catalogNumber: BMNHE1722108; sex: Male; **Taxon:** scientificName: Micropygomyia (Micropygomyia) cayennensis braci (Lewis, 1967); **Location:** country: Cayman Islands; stateProvince: Cayman Brac; locality: Stake Bay; **Event:** eventDate: 12-16-64; eventRemarks: http://phlebotominaenhmtypes.myspecies.info/node/227; **Record Level:** institutionCode: NHMUK; basisOfRecord: Preserved Specimen
- **Type status:** Paratype. **Occurrence:** catalogNumber: BMNHE1722109; sex: Male; **Taxon:** scientificName: Micropygomyia (Micropygomyia) cayennensis braci (Lewis, 1967); **Location:** country: Cayman Islands; stateProvince: Cayman Brac; locality: Stake Bay; **Event:** eventDate: 12-16-64; eventRemarks: http://phlebotominaenhmtypes.myspecies.info/node/228; **Record Level:** institutionCode: NHMUK; basisOfRecord: Preserved Specimen
- **Type status:** Paratype. **Occurrence:** catalogNumber: BMNHE1722110; sex: Female; **Taxon:** scientificName: Micropygomyia (Micropygomyia) cayennensis braci (Lewis, 1967); **Location:** country: Cayman Islands; stateProvince: Cayman Brac; locality: Bamboo Bay; **Event:** eventDate: 12-18-64; eventRemarks: http://phlebotominaenhmtypes.myspecies.info/node/229; **Record Level:** institutionCode: NHMUK; basisOfRecord: Preserved Specimen
- **Type status:** Paratype. **Occurrence:** catalogNumber: BMNHE1722111; sex: Male; **Taxon:** scientificName: Micropygomyia (Micropygomyia) cayennensis braci (Lewis, 1967); **Location:** country: Cayman Islands; stateProvince: Cayman Brac; locality: Bamboo Bay; **Event:** eventDate: 12-18-64; eventRemarks: http://phlebotominaenhmtypes.myspecies.info/node/230; **Record Level:** institutionCode: NHMUK; basisOfRecord: Preserved Specimen
- **Type status:** Paratype. **Occurrence:** catalogNumber: BMNHE1722112; sex: Female; **Taxon:** scientificName: Micropygomyia (Micropygomyia) cayennensis braci (Lewis, 1967); **Location:** country: Cayman Islands; stateProvince: Cayman Brac; locality: Stake Bay; **Event:** eventDate: 12-16-64; eventRemarks: http://phlebotominaenhmtypes.myspecies.info/node/231; **Record Level:** institutionCode: NHMUK; basisOfRecord: Preserved Specimen
- **Type status:** Paratype. **Occurrence:** catalogNumber: BMNHE1722113; sex: Male; **Taxon:** scientificName: Micropygomyia (Micropygomyia) cayennensis braci (Lewis, 1967); **Location:** country: Cayman Islands; stateProvince: Cayman Brac; locality: Bamboo Bay; **Event:** eventDate: 12-18-64; eventRemarks: http://phlebotominaenhmtypes.myspecies.info/node/232; **Record Level:** institutionCode: NHMUK; basisOfRecord: Preserved Specimen

##### Distribution

Cayman Islands

##### Notes

Valid species in Micropygomyia (Micropygomyia).

#### Micropygomyia cayennensis hispaniolae

Fairchild & Trapido, 1950

Phlebotomus cayennensis hispaniolae Fairchild & Trapido, 1950 (Fairchild and Trapido 1950)

##### Materials

- **Type status:** Paratype. **Occurrence:** catalogNumber: BMNHE1722029; sex: Female; **Taxon:** scientificName: Micropygomyia (Micropygomyia) cayennensis hispaniolae (Fairchild & Trapido, 1950); **Location:** country: Haiti; stateProvince: Nord; locality: La Voute, 4.8 miles from Cap-Haïtien on Limbe Rd.; **Event:** eventDate: 06-09-49; eventRemarks: http://phlebotominaenhmtypes.myspecies.info/node/148; **Record Level:** institutionCode: NHMUK; basisOfRecord: Preserved Specimen
- **Type status:** Paratype. **Occurrence:** catalogNumber: BMNHE1722030; sex: Male; **Taxon:** scientificName: Micropygomyia (Micropygomyia) cayennensis hispaniolae (Fairchild & Trapido, 1950); **Location:** country: Haiti; stateProvince: Nord; municipality: le Cap-Haïtien; locality: Milot, le Cap-Haïtien; **Event:** eventDate: 06-09-49; eventRemarks: http://phlebotominaenhmtypes.myspecies.info/node/149; **Record Level:** institutionCode: NHMUK; basisOfRecord: Preserved Specimen
- **Type status:** Paratype. **Occurrence:** catalogNumber: BMNHE1722031; sex: Female; **Taxon:** scientificName: Micropygomyia (Micropygomyia) cayennensis hispaniolae (Fairchild & Trapido, 1950); **Location:** country: Dominican Republic; stateProvince: Hato Mayor; municipality: Sabana de la Mar; locality: Cacao plantation 6 km South of Sabana de la Mar; **Event:** eventDate: 06-12-49; eventRemarks: http://phlebotominaenhmtypes.myspecies.info/node/150; **Record Level:** institutionCode: NHMUK; basisOfRecord: Preserved Specimen
- **Type status:** Paratype. **Occurrence:** catalogNumber: BMNHE1722032; sex: Female; **Taxon:** scientificName: Micropygomyia (Micropygomyia) cayennensis hispaniolae (Fairchild & Trapido, 1950); **Location:** country: Haiti; stateProvince: Nord; municipality: le Cap-Haïtien; locality: Ruins of Pauline Bonaparte's palace East of town; **Event:** eventDate: 06-09-49; eventRemarks: http://phlebotominaenhmtypes.myspecies.info/node/151; **Record Level:** institutionCode: NHMUK; basisOfRecord: Preserved Specimen
- **Type status:** Paratype. **Occurrence:** catalogNumber: BMNHE1722033; sex: Male; **Taxon:** scientificName: Micropygomyia (Micropygomyia) cayennensis hispaniolae (Fairchild & Trapido, 1950); **Location:** country: Haiti; stateProvince: Nord; municipality: le Cap-Haïtien; locality: Ruins of Pauline Bonaparte's palace East of town; **Event:** eventDate: 06-09-49; eventRemarks: http://phlebotominaenhmtypes.myspecies.info/node/152; **Record Level:** institutionCode: NHMUK; basisOfRecord: Preserved Specimen
- **Type status:** Paratype. **Occurrence:** catalogNumber: BMNHE1722034; sex: Male; **Taxon:** scientificName: Micropygomyia (Micropygomyia) cayennensis hispaniolae (Fairchild & Trapido, 1950); **Location:** country: Dominican Republic; stateProvince: Santiago; locality: Santiago area; **Event:** eventDate: 06-14-49; eventRemarks: http://phlebotominaenhmtypes.myspecies.info/node/153; **Record Level:** institutionCode: NHMUK; basisOfRecord: Preserved Specimen

##### Distribution

Dominican Republic, Haiti

##### Notes

Valid species in Micropygomyia (Micropygomyia).

#### Micropygomyia cayennensis jamaicensis

Fairchild & Trapido, 1950

Phlebotomus cayennensis jamaicensis Fairchild & Trapido, 1950 (Fairchild and Trapido 1950)

##### Materials

- **Type status:** Paratype. **Occurrence:** catalogNumber: BMNHE1722037; sex: Male; **Taxon:** scientificName: Micropygomyia (Micropygomyia) cayennensis jamaicensis (Fairchild & Trapido, 1950); **Location:** country: Jamaica; stateProvince: Kingston; locality: Rockfort, St Andrew parish, Kingston; **Event:** eventDate: 05-20-49; eventRemarks: http://phlebotominaenhmtypes.myspecies.info/node/156; **Record Level:** institutionCode: NHMUK; basisOfRecord: Preserved Specimen

##### Distribution

Jamaica

##### Notes

Valid species in Micropygomyia (Micropygomyia).

#### Micropygomyia cayennensis maciasi

Fairchild & Hertig, 1948

Phlebotomus cayennensis maciasi Fairchild & Hertig, 1948 (Fairchild and Hertig 1948)

##### Materials

- **Type status:** Paratype. **Occurrence:** catalogNumber: BMNHE1722035; sex: Female; **Taxon:** scientificName: Micropygomyia (Micropygomyia) cayennensis maciasi (Fairchild & Hertig, 1948); **Location:** country: Mexico; stateProvince: México; municipality: Zumpango; locality: Zumpango; **Event:** eventRemarks: http://phlebotominaenhmtypes.myspecies.info/node/154; **Record Level:** institutionCode: NHMUK; basisOfRecord: Preserved Specimen
- **Type status:** Paratype. **Occurrence:** catalogNumber: BMNHE1722036; sex: Female; **Taxon:** scientificName: Micropygomyia (Micropygomyia) cayennensis maciasi (Fairchild & Hertig, 1948); **Location:** country: Guatemala; stateProvince: Escuintla; municipality: San José; locality: San José; **Event:** eventDate: 06-03-45; eventRemarks: http://phlebotominaenhmtypes.myspecies.info/node/155; **Record Level:** institutionCode: NHMUK; basisOfRecord: Preserved Specimen

##### Distribution

Belize, Guatemala, Mexico

##### Notes

Valid species in Micropygomyia (Micropygomyia).

#### Micropygomyia cayennensis puertoricensis

Fairchild & Hertig, 1948

Phlebotomus cayennensis puertoricensis Fairchild & Hertig, 1948 (Fairchild and Hertig 1948)

##### Materials

- **Type status:** Paratype. **Occurrence:** catalogNumber: BMNHE1722040; sex: Female; **Taxon:** scientificName: Micropygomyia (Micropygomyia) cayennensis puertoricensis (Fairchild & Hertig, 1948); **Location:** country: Puerto Rico; stateProvince: Lares; locality: Lares; **Event:** eventDate: 07-03-47; eventRemarks: http://phlebotominaenhmtypes.myspecies.info/node/159; **Record Level:** institutionCode: NHMUK; basisOfRecord: Preserved Specimen

##### Distribution

Puerto Rico

##### Notes

Valid species in Micropygomyia (Micropygomyia).

#### Psathyromyia coutinhoi

Mangabeira, 1942

Flebotomus coutinhoi Mangabeira, 1942 (Mangabeira 1942)

##### Materials

- **Type status:** Paratype. **Occurrence:** catalogNumber: BMNHE1721985; sex: Male; **Taxon:** scientificName: Psathyromyia (Forattiniella) coutinhoi (Mangabeira, 1942); **Location:** country: Brazil; stateProvince: Pará; municipality: Belém; locality: Asura; **Event:** year: 1940; month: 8; eventRemarks: http://phlebotominaenhmtypes.myspecies.info/node/104; **Record Level:** institutionCode: NHMUK; basisOfRecord: Preserved Specimen

##### Distribution

Bolivia, Brazil, Peru

##### Notes

Valid species in Psathyromyia (Forattiniella).

#### Micropygomyia cubensis

Fairchild & Trapido, 1950

Phlebotomus cubensis Fairchild & Trapido, 1950 (Fairchild and Trapido 1950)

##### Materials

- **Type status:** Paratype. **Occurrence:** catalogNumber: BMNHE1722041; sex: Female; **Taxon:** scientificName: Micropygomyia (Micropygomyia) cubensis (Fairchild & Trapido, 1950); **Location:** country: Cuba; stateProvince: Camagüey; locality: Camagüey; **Event:** eventDate: 05-30-49; eventRemarks: http://phlebotominaenhmtypes.myspecies.info/node/160; **Record Level:** institutionCode: NHMUK; basisOfRecord: Preserved Specimen
- **Type status:** Paratype. **Occurrence:** catalogNumber: BMNHE1722042; sex: Male; **Taxon:** scientificName: Micropygomyia (Micropygomyia) cubensis (Fairchild & Trapido, 1950); **Location:** country: Cuba; stateProvince: Pinar del Río; municipality: Viñales; locality: San Vincente, Viñales; **Event:** eventDate: 06-04-49; eventRemarks: http://phlebotominaenhmtypes.myspecies.info/node/161; **Record Level:** institutionCode: NHMUK; basisOfRecord: Preserved Specimen
- **Type status:** Paratype. **Occurrence:** catalogNumber: BMNHE1722114; sex: Female; **Taxon:** scientificName: Micropygomyia (Micropygomyia) cubensis (Fairchild & Trapido, 1950); **Location:** country: Cuba; stateProvince: Camagüey; locality: spelling on slide "Camaguay"; **Event:** eventDate: 05-30-49; eventRemarks: http://phlebotominaenhmtypes.myspecies.info/node/233; **Record Level:** institutionCode: NHMUK; basisOfRecord: Preserved Specimen
- **Type status:** Paratype. **Occurrence:** catalogNumber: BMNHE1722115; **Taxon:** scientificName: Micropygomyia (Micropygomyia) cubensis (Fairchild & Trapido, 1950); **Location:** country: Cuba; stateProvince: Matanzas; locality: Ceiba Mocha; **Event:** eventDate: 06-02-49; eventRemarks: http://phlebotominaenhmtypes.myspecies.info/node/234; **Record Level:** institutionCode: NHMUK; basisOfRecord: Preserved Specimen
- **Type status:** Paratype. **Occurrence:** catalogNumber: BMNHE1722116; sex: Male; **Taxon:** scientificName: Micropygomyia (Micropygomyia) cubensis (Fairchild & Trapido, 1950); **Location:** country: Cuba; stateProvince: Camagüey; locality: spelling on slide "Camaguay"; **Event:** eventDate: 05-30-49; eventRemarks: http://phlebotominaenhmtypes.myspecies.info/node/235; **Record Level:** institutionCode: NHMUK; basisOfRecord: Preserved Specimen

##### Distribution

Cuba, United States of America

##### Notes

Valid species in Micropygomyia (Micropygomyia).

#### Psathyromyia dasymera

Fairchild & Hertig, 1961

Phlebotomus dasymerus Fairchild & Hertig, 1961 (Fairchild and Hertig 1961)

##### Materials

- **Type status:** Paratype. **Occurrence:** catalogNumber: BMNHE1251321; sex: Male; **Taxon:** scientificName: Psathyromyia (Psathyromyia) dasymera (Fairchild & Hertig, 1961); **Location:** country: Panama; stateProvince: Colón; locality: Juan Mina; **Event:** eventDate: 11-29-49; eventRemarks: http://phlebotominaenhmtypes.myspecies.info/node/93; **Record Level:** institutionCode: NHMUK; basisOfRecord: Preserved Specimen
- **Type status:** Paratype. **Occurrence:** catalogNumber: BMNHE1251322; sex: Male; **Taxon:** scientificName: Psathyromyia (Psathyromyia) dasymera (Fairchild & Hertig, 1961); **Location:** country: Panama; stateProvince: Panamá; locality: Rio Majé, Charco el Toro; **Event:** eventDate: 03-22-50; eventRemarks: http://phlebotominaenhmtypes.myspecies.info/node/94; **Record Level:** institutionCode: NHMUK; basisOfRecord: Preserved Specimen
- **Type status:** Paratype. **Occurrence:** catalogNumber: BMNHE1251323; sex: Female; **Taxon:** scientificName: Psathyromyia (Psathyromyia) dasymera (Fairchild & Hertig, 1961); **Location:** country: Panama; locality: Canal zone, Camp Piña; **Event:** eventDate: 03-06-54; eventRemarks: http://phlebotominaenhmtypes.myspecies.info/node/95; **Record Level:** institutionCode: NHMUK; basisOfRecord: Preserved Specimen
- **Type status:** Paratype. **Occurrence:** catalogNumber: BMNHE1251324; sex: Female; **Taxon:** scientificName: Psathyromyia (Psathyromyia) dasymera (Fairchild & Hertig, 1961); **Location:** country: Panama; stateProvince: Colón; locality: Mojinga swamp; **Event:** eventDate: 07-24-54; eventRemarks: http://phlebotominaenhmtypes.myspecies.info/node/96; **Record Level:** institutionCode: NHMUK; basisOfRecord: Preserved Specimen

##### Distribution

Belize, Brazil, Colombia, Costa Rica, Ecuador, Mexico, Nicaragua, Panama, Venezuela

##### Notes

Valid species in Psathyromyia (Psathyromyia).

#### Dampfomyia del-pozoi

Vargas & Díaz-Nájera, 1953

Phlebotomus del-pozoi Vargas & Díaz-Nájera, 1953 (Vargas and Díaz Nájera 1953)

##### Materials

- **Type status:** Paratype. **Occurrence:** catalogNumber: BMNHE1722142; sex: Female; **Taxon:** scientificName: *Dampfomyia delpozoi* (Vargas & Díaz-Nájera, 1953); **Location:** country: Mexico; stateProvince: Chiapas; locality: Finca El Vergel; **Event:** eventDate: 05-28-35; eventRemarks: http://phlebotominaenhmtypes.myspecies.info/node/261; **Record Level:** institutionCode: NHMUK; basisOfRecord: Preserved Specimen
- **Type status:** Paratype. **Occurrence:** catalogNumber: BMNHE1722143; sex: Female; **Taxon:** scientificName: *Dampfomyia delpozoi* (Vargas & Díaz-Nájera, 1953); **Location:** country: Mexico; stateProvince: Chiapas; locality: Finca El Vergel; **Event:** eventDate: 05-28-35; eventRemarks: http://phlebotominaenhmtypes.myspecies.info/node/262; **Record Level:** institutionCode: NHMUK; basisOfRecord: Preserved Specimen

##### Distribution

Belize, Guatemala, Mexico

##### Notes

Valid species in *Dampfomyia*.

#### Micropygomyia duppyorum

Fairchild & Trapido, 1950

Phlebotomus duppyorum Fairchild & Trapido, 1950 (Fairchild and Trapido 1950)

##### Materials

- **Type status:** Paratype. **Occurrence:** catalogNumber: BMNHE1722070; sex: Female; **Taxon:** scientificName: Micropygomyia (Micropygomyia) duppyorum (Fairchild & Trapido, 1950); **Location:** country: Jamaica; stateProvince: Manchester; locality: Melrose Hill, nr. Williams Field; **Event:** eventDate: 05-25-49; eventRemarks: http://phlebotominaenhmtypes.myspecies.info/node/189; **Record Level:** institutionCode: NHMUK; basisOfRecord: Preserved Specimen
- **Type status:** Paratype. **Occurrence:** catalogNumber: BMNHE1722071; sex: Male; **Taxon:** scientificName: Micropygomyia (Micropygomyia) duppyorum (Fairchild & Trapido, 1950); **Location:** country: Jamaica; stateProvince: Manchester; locality: Melrose Hill, nr. Williams Field; **Event:** eventDate: 05-25-49; eventRemarks: http://phlebotominaenhmtypes.myspecies.info/node/190; **Record Level:** institutionCode: NHMUK; basisOfRecord: Preserved Specimen
- **Type status:** Paratype. **Occurrence:** catalogNumber: BMNHE1722117; sex: Female; **Taxon:** scientificName: Micropygomyia (Micropygomyia) duppyorum (Fairchild & Trapido, 1950); **Location:** country: Jamaica; stateProvince: Manchester; locality: Melrose Hill, nr. Williams Field.; **Event:** eventDate: 05-25-49; eventRemarks: http://phlebotominaenhmtypes.myspecies.info/node/236; **Record Level:** institutionCode: NHMUK; basisOfRecord: Preserved Specimen
- **Type status:** Paratype. **Occurrence:** catalogNumber: BMNHE1722118; sex: Male; **Taxon:** scientificName: Micropygomyia (Micropygomyia) duppyorum (Fairchild & Trapido, 1950); **Location:** country: Jamaica; stateProvince: Saint Catherine; locality: nr. Springvale; **Event:** eventDate: 05-21-49; eventRemarks: http://phlebotominaenhmtypes.myspecies.info/node/237; **Record Level:** institutionCode: NHMUK; basisOfRecord: Preserved Specimen
- **Type status:** Paratype. **Occurrence:** catalogNumber: BMNHE1722119; **Taxon:** scientificName: Micropygomyia (Micropygomyia) duppyorum (Fairchild & Trapido, 1950); **Location:** country: Jamaica; stateProvince: Manchester; locality: Manchester area; **Event:** eventDate: 05-25-49; eventRemarks: http://phlebotominaenhmtypes.myspecies.info/node/238; **Record Level:** institutionCode: NHMUK; basisOfRecord: Preserved Specimen

##### Distribution

Jamaica

##### Notes

Valid species in Micropygomyia (Micropygomyia).

#### Pressatia dysponeta

Fairchild & Hertig, 1952

Phlebotomus dysponeta Fairchild & Hertig, 1952 (Fairchild and Hertig 1952)

##### Materials

- **Type status:** Paratype. **Occurrence:** catalogNumber: BMNHE1722075; sex: Female; **Taxon:** scientificName: *Pressatia dysponeta* (Fairchild & Hertig, 1952); **Location:** country: Panama; stateProvince: Panamá; locality: Pacora; **Event:** eventDate: 08-13-50; eventRemarks: http://phlebotominaenhmtypes.myspecies.info/node/194; **Record Level:** institutionCode: NHMUK; basisOfRecord: Preserved Specimen
- **Type status:** Paratype. **Occurrence:** catalogNumber: BMNHE1722076; sex: Male; **Taxon:** scientificName: *Pressatia dysponeta* (Fairchild & Hertig, 1952); **Location:** country: Panama; stateProvince: Panamá; locality: Pacora; **Event:** eventDate: 08-13-50; eventRemarks: http://phlebotominaenhmtypes.myspecies.info/node/195; **Record Level:** institutionCode: NHMUK; basisOfRecord: Preserved Specimen

##### Distribution

Brazil, Colombia, Costa Rica, Ecuador, Panama, Venezuela

##### Notes

Valid species in *Pressatia*.

#### Pintomyia evansi

Nuñez-Tovar, 1924

Phlebotomus evansi Nuñez-Tovar, 1924 (Nuñez-Tovar 1924)

##### Materials

- **Type status:** Syntype. **Occurrence:** catalogNumber: BMNHE1722089; sex: Male; **Taxon:** scientificName: Pintomyia (Pifanomyia) evansi (Nuñez-Tovar, 1924); **Location:** country: Venezuela; **Event:** year: 1922; eventRemarks: http://phlebotominaenhmtypes.myspecies.info/node/208; **Record Level:** institutionCode: NHMUK; basisOfRecord: Preserved Specimen
- **Type status:** Syntype. **Occurrence:** catalogNumber: BMNHE1722091; sex: Male; **Taxon:** scientificName: Pintomyia (Pifanomyia) evansi (Nuñez-Tovar, 1924); **Location:** country: Venezuela; **Event:** year: 1922; eventRemarks: http://phlebotominaenhmtypes.myspecies.info/node/210; **Record Level:** institutionCode: NHMUK; basisOfRecord: Preserved Specimen

##### Distribution

Colombia, Costa Rica, El Salvador, Guatemala, Honduras, Mexico, Nicaragua, Peru, Venezuela

##### Notes

Valid species in Pintomyia (Pifanomyia).

#### Trichophoromyia flochi

Abonnenc & Chassignet, 1948

Phlebotomus flochi Abonnenc & Chassignet, 1948 (Abonnenc and Chassignet 1948)

##### Materials

- **Type status:** Holotype. **Occurrence:** catalogNumber: BMNHE1722138; sex: Male; **Taxon:** scientificName: *Trichophoromyia flochi* (Abonnenc & Chassignet, 1948); **Location:** country: French Guiana; locality: Baduel; **Event:** year: 1947; month: 12; eventRemarks: http://phlebotominaenhmtypes.myspecies.info/node/257; **Record Level:** institutionCode: NHMUK; basisOfRecord: Preserved Specimen

##### Distribution

Brazil, French Guiana

##### Notes

Valid species in *Trichophoromyia*.

#### Evandromyia gasti

Sherlock, 1962

Phlebotomus gasti Sherlock, 1962 (Sherlock 1962)

##### Materials

- **Type status:** Paratype. **Occurrence:** catalogNumber: BMNHE1722096; sex: Male; **Taxon:** scientificName: Evandromyia (Aldamyia) gasti (Sherlock, 1962); acceptedNameUsage: Evandromyia (Aldamyia) walkeri (Newstead, 1914); **Location:** country: Colombia; stateProvince: Santander; municipality: San Vicente de Chucurí; locality: Soledad, San Vicente de Chucurí; **Event:** eventDate: 08-16-74; eventRemarks: http://phlebotominaenhmtypes.myspecies.info/node/215; **Record Level:** institutionCode: NHMUK; basisOfRecord: Preserved Specimen
- **Type status:** Paratype. **Occurrence:** catalogNumber: BMNHE1722097; sex: Male; **Taxon:** scientificName: Evandromyia (Aldamyia) gasti (Sherlock, 1962); acceptedNameUsage: Evandromyia (Aldamyia) walkeri (Newstead, 1914); **Location:** country: Colombia; stateProvince: Santander; municipality: San Vicente de Chucurí; locality: Soledad, San Vicente de Chucurí; **Event:** eventDate: 16/08/44; eventRemarks: http://phlebotominaenhmtypes.myspecies.info/node/216; **Record Level:** institutionCode: NHMUK; basisOfRecord: Preserved Specimen

##### Distribution

Bolivia, Brazil, Colombia, Ecuador, French Guiana, Panama, Peru, Trinidad and Tobago, Venezuela

##### Notes

Synonym of Evandromyia (Aldamyia) walkeri (Newstead, 1914). Synonymised by Young 1979.

#### Lutzomyia gonzaloi

Ogusuku, Canales & Pérez, 1997

Lutzomyia gonzaloi Ogusuku, Canales & Pérez, 1997 (Ogusuku et al. 1997)

##### Materials

- **Type status:** Paratype. **Occurrence:** catalogNumber: BMNHE1722012; sex: Male; **Taxon:** scientificName: Lutzomyia (Helcocyrtomyia) gonzaloi Ogusuku, Canales & Pérez, 1997; **Location:** country: Peru; stateProvince: Huánuco; municipality: Huamalíes; locality: Monzon, Paucacu; **Event:** eventDate: 05-22-97; eventRemarks: http://phlebotominaenhmtypes.myspecies.info/node/131; **Record Level:** institutionCode: NHMUK; basisOfRecord: Preserved Specimen
- **Type status:** Paratype. **Occurrence:** catalogNumber: BMNHE1722013; sex: Female; **Taxon:** scientificName: Lutzomyia (Helcocyrtomyia) gonzaloi Ogusuku, Canales & Pérez, 1998; **Location:** country: Peru; stateProvince: Huánuco; municipality: Huamalíes; locality: Monzon, Paucacu; **Event:** eventDate: 05-22-97; eventRemarks: http://phlebotominaenhmtypes.myspecies.info/node/132; **Record Level:** institutionCode: NHMUK; basisOfRecord: Preserved Specimen

##### Distribution

Peru

##### Notes

Valid species in Lutzomyia (Helcocyrtomyia).

#### Migonemyia hansoni

Fairchild & Hertig, 1961

Phlebotomus hansoni Fairchild & Hertig, 1961 (Fairchild and Hertig 1961)

##### Materials

- **Type status:** Paratype. **Occurrence:** catalogNumber: BMNHE1721997; sex: Female; **Taxon:** scientificName: Migonemyia (Blancasmyia) hansoni (Fairchild & Hertig, 1961); acceptedNameUsage: Migonemyia (Blancasmyia) gorbitzi (Blancas, 1960); **Location:** country: Panama; stateProvince: Bocas del Toro; locality: Almirante; **Event:** eventDate: 06-19-51; eventRemarks: http://phlebotominaenhmtypes.myspecies.info/node/116; **Record Level:** institutionCode: NHMUK; basisOfRecord: Preserved Specimen
- **Type status:** Paratype. **Occurrence:** catalogNumber: BMNHE1721998; sex: Female; **Taxon:** scientificName: Migonemyia (Blancasmyia) hansoni (Fairchild & Hertig, 1961); acceptedNameUsage: Migonemyia (Blancasmyia) gorbitzi (Blancas, 1960); **Location:** country: Panama; stateProvince: Bocas del Toro; locality: Almirante; **Event:** eventDate: 06-19-51; eventRemarks: http://phlebotominaenhmtypes.myspecies.info/node/117; **Record Level:** institutionCode: NHMUK; basisOfRecord: Preserved Specimen

##### Distribution

Colombia, Costa Rica, Ecuador, Panama, Peru

##### Notes

Synonym of Migonemyia (Blancasmyia) gorbitzi (Blancas, 1959) Blancas 1959. Synonymised by Christensen and Rutledge 1973.

#### Lutzomyia hartmanni

Fairchild & Hertig, 1957

Phlebotomus hartmanni Fairchild & Hertig, 1957 (Fairchild and Hertig 1957)

##### Materials

- **Type status:** Paratype. **Occurrence:** catalogNumber: BMNHE1722007; sex: Male; **Taxon:** scientificName: Lutzomyia (Helcocyrtomyia) hartmanni (Fairchild & Hertig, 1957); **Location:** country: Panama; locality: Cerro Campana; **Event:** eventDate: 04-24-52; eventRemarks: http://phlebotominaenhmtypes.myspecies.info/node/126; **Record Level:** institutionCode: NHMUK; basisOfRecord: Preserved Specimen
- **Type status:** Paratype. **Occurrence:** catalogNumber: BMNHE1722008; sex: Female; **Taxon:** scientificName: Lutzomyia (Helcocyrtomyia) hartmanni (Fairchild & Hertig, 1957); **Location:** country: Panama; locality: Cerro Campana; **Event:** eventDate: 02-10-51; eventRemarks: http://phlebotominaenhmtypes.myspecies.info/node/127; **Record Level:** institutionCode: NHMUK; basisOfRecord: Preserved Specimen

##### Distribution

Colombia, Costa Rica, Ecuador, Mexico, Panama, Peru

##### Notes

Valid species in Lutzomyia (Helcocyrtomyia).

#### Evandromyia inpai

Young & Arias, 1977

Lutzomyia inpai Young & Arias, 1977 (Young and Arias 1977)

##### Materials

- **Type status:** Paratype. **Occurrence:** catalogNumber: BMNHE1721994; sex: Female; **Taxon:** scientificName: Evandromyia (Evandromyia) inpai (Young & Arias, 1977); **Location:** country: Brazil; stateProvince: Amazonas State; locality: Manaus, Estrada Torquato Tapajos, km 54 (east of Manaus); **Event:** eventDate: 10-21-75; eventRemarks: http://phlebotominaenhmtypes.myspecies.info/node/113; **Record Level:** institutionCode: NHMUK; basisOfRecord: Preserved Specimen
- **Type status:** Paratype. **Occurrence:** catalogNumber: BMNHE1721995; sex: Male; **Taxon:** scientificName: Evandromyia (Evandromyia) inpai (Young & Arias, 1977); **Location:** country: Brazil; stateProvince: Amazonas State; locality: Manaus, Estrada Torquato Tapajos, km 54 (east of Manaus); **Event:** eventDate: 10-21-75; eventRemarks: http://phlebotominaenhmtypes.myspecies.info/node/114; **Record Level:** institutionCode: NHMUK; basisOfRecord: Preserved Specimen

##### Distribution

Brazil, Venezuela

##### Notes

Valid species in Evandromyia (Evandromyia).

#### Dampfomyia isovespertilionis

Fairchild & Hertig, 1958

Phlebotomus isovespertilionis Fairchild & Hertig, 1958 (Fairchild and Hertig 1958a)

##### Materials

- **Type status:** Paratype. **Occurrence:** catalogNumber: BMNHE1721986; sex: Male; **Taxon:** scientificName: Dampfomyia (Coromyia) isovespertilionis (Fairchild & Hertig, 1958); **Location:** country: Panama; **Event:** eventDate: 06-20-56; eventRemarks: http://phlebotominaenhmtypes.myspecies.info/node/105; **Record Level:** institutionCode: NHMUK; basisOfRecord: Preserved Specimen
- **Type status:** Paratype. **Occurrence:** catalogNumber: BMNHE1721987; sex: Male; **Taxon:** scientificName: Dampfomyia (Coromyia) isovespertilionis (Fairchild & Hertig, 1958); **Location:** country: Panama; **Event:** eventDate: 06-20-56; eventRemarks: http://phlebotominaenhmtypes.myspecies.info/node/106; **Record Level:** institutionCode: NHMUK; basisOfRecord: Preserved Specimen
- **Type status:** Paratype. **Occurrence:** catalogNumber: BMNHE1721988; sex: Male; **Taxon:** scientificName: Dampfomyia (Coromyia) isovespertilionis (Fairchild & Hertig, 1958); **Location:** country: Panama; **Event:** eventDate: 06-20-56; eventRemarks: http://phlebotominaenhmtypes.myspecies.info/node/107; **Record Level:** institutionCode: NHMUK; basisOfRecord: Preserved Specimen
- **Type status:** Paratype. **Occurrence:** catalogNumber: BMNHE1721989; sex: Male; **Taxon:** scientificName: Dampfomyia (Coromyia) isovespertilionis (Fairchild & Hertig, 1958); **Location:** country: Panama; **Event:** eventDate: 06-20-56; eventRemarks: http://phlebotominaenhmtypes.myspecies.info/node/108; **Record Level:** institutionCode: NHMUK; basisOfRecord: Preserved Specimen
- **Type status:** Paratype. **Occurrence:** catalogNumber: BMNHE1721990; sex: Male; **Taxon:** scientificName: Dampfomyia (Coromyia) isovespertilionis (Fairchild & Hertig, 1958); **Location:** country: Panama; **Event:** eventDate: 06-20-56; eventRemarks: http://phlebotominaenhmtypes.myspecies.info/node/109; **Record Level:** institutionCode: NHMUK; basisOfRecord: Preserved Specimen
- **Type status:** Paratype. **Occurrence:** catalogNumber: BMNHE1722047; sex: Male; **Taxon:** scientificName: Dampfomyia (Coromyia) isovespertilionis (Fairchild & Hertig, 1958); **Location:** country: Panama; locality: K-19; **Event:** eventDate: 07-24-56; eventRemarks: http://phlebotominaenhmtypes.myspecies.info/node/166; **Record Level:** institutionCode: NHMUK; basisOfRecord: Preserved Specimen

##### Distribution

Colombia, Costa Rica, Panama

##### Notes

Valid species in Dampfomyia (Coromyia).

#### Psychodopygus lainsoni

Fraiha & Ward, 1974

Psychodopygus lainsoni Fraiha & Ward, 1974 (Fraiha and Ward 1974)

##### Materials

- **Type status:** Paratype. **Occurrence:** catalogNumber: BMNHE1722077; sex: Female; **Taxon:** scientificName: *Psychodopygus lainsoni* (Fraiha & Ward, 1974); **Location:** country: Brazil; stateProvince: Pará; locality: km 46 Altamira to Itaituba road, Transamazonia highway; **Event:** eventDate: 08-21-71; eventRemarks: http://phlebotominaenhmtypes.myspecies.info/node/196; **Record Level:** institutionCode: NHMUK; basisOfRecord: Preserved Specimen
- **Type status:** Paratype. **Occurrence:** catalogNumber: BMNHE1722078; sex: Male; **Taxon:** scientificName: *Psychodopygus lainsoni* (Fraiha & Ward, 1974); **Location:** country: Brazil; stateProvince: Pará; locality: km 46 Altamira to Itaituba road, Transamazonia highway; **Event:** year: 1971; eventRemarks: http://phlebotominaenhmtypes.myspecies.info/node/197; **Record Level:** institutionCode: NHMUK; basisOfRecord: Preserved Specimen
- **Type status:** Paratype. **Occurrence:** catalogNumber: BMNHE1722079; sex: Female; **Taxon:** scientificName: *Psychodopygus lainsoni* (Fraiha & Ward, 1974); **Location:** country: Brazil; stateProvince: Pará; locality: km 46 Altamira to Itaituba road, Transamazonia highway; **Event:** eventDate: 08-21-71; eventRemarks: http://phlebotominaenhmtypes.myspecies.info/node/198; **Record Level:** institutionCode: NHMUK; basisOfRecord: Preserved Specimen
- **Type status:** Paratype. **Occurrence:** catalogNumber: BMNHE1722080; sex: Male; **Taxon:** scientificName: *Psychodopygus lainsoni* (Fraiha & Ward, 1974); **Location:** country: Brazil; stateProvince: Pará; locality: km 46 Altamira to Itaituba road, Transamazonia highway; **Event:** eventRemarks: http://phlebotominaenhmtypes.myspecies.info/node/199; **Record Level:** institutionCode: NHMUK; basisOfRecord: Preserved Specimen

##### Distribution

Bolivia, Brazil, Peru

##### Notes

Valid species in *Psychodopygus*.

#### Micropygomyia lewisi

Feliciangeli, Ordoñez & Fernández, 1984

Lutzomyia lewisi Feliciangeli, Ordoñez & Fernández, 1984 (Feliciangeli et al. 1984a)

##### Materials

- **Type status:** Holotype. **Occurrence:** catalogNumber: BMNHE1721984; sex: Female; **Taxon:** scientificName: Micropygomyia (Micropygomyia) lewisi (Feliciangeli, Ordoñez & Fernández, 1984); **Location:** country: Venezuela; stateProvince: Cojedes; locality: La Vāquira; **Event:** eventDate: 06-20-77; eventRemarks: http://phlebotominaenhmtypes.myspecies.info/node/103; **Record Level:** institutionCode: NHMUK; basisOfRecord: Preserved Specimen

##### Distribution

Venezuela

##### Notes

Valid species in Micropygomyia (Micropygomyia).

#### Warileya lumbrerasi

Ogosuku, Perez, Davies & Villaseca, 1996

Warileya lumbrerasi Ogosuku, Perez, Davies & Villaseca, 1996 (Ogusuku et al. 1996)

##### Materials

- **Type status:** Paratype. **Occurrence:** catalogNumber: BMNHE1721980; sex: Female; **Taxon:** scientificName: *Warileya lumbrerasi* Ogosuku, Perez, Davies & Villaseca, 1996; **Location:** country: Peru; stateProvince: Piura; municipality: Huancabamba; locality: El Higueron; **Event:** eventDate: 02-21-93; eventRemarks: http://phlebotominaenhmtypes.myspecies.info/node/99; **Record Level:** institutionCode: NHMUK; basisOfRecord: Preserved Specimen
- **Type status:** Paratype. **Occurrence:** catalogNumber: BMNHE1721981; sex: Male; **Taxon:** scientificName: *Warileya lumbrerasi* Ogosuku, Perez, Davies & Villaseca, 1996; **Location:** country: Peru; stateProvince: Piura; municipality: Huancabamba; locality: El Higueron; **Event:** eventDate: 05-23-92; eventRemarks: http://phlebotominaenhmtypes.myspecies.info/node/100; **Record Level:** institutionCode: NHMUK; basisOfRecord: Preserved Specimen

##### Distribution

Peru

##### Notes

Valid species in *Warileya*.

#### Lutzomyia monzonensis

Ogusuku, Canales & Pérez, 1997

Lutzomyia monzonensis Ogusuku, Canales & Pérez, 1997 (Ogusuku et al. 1997)

##### Materials

- **Type status:** Paratype. **Occurrence:** catalogNumber: BMNHE1722014; sex: Female; **Taxon:** scientificName: Lutzomyia (Helcocyrtomyia) monzonensis Ogusuku, Canales & Pérez, 1997; **Location:** country: Peru; stateProvince: Huánuco; municipality: Huamalíes; locality: Monzon, Paucacu; **Event:** eventDate: 05-22-97; eventRemarks: http://phlebotominaenhmtypes.myspecies.info/node/133; **Record Level:** institutionCode: NHMUK; basisOfRecord: Preserved Specimen
- **Type status:** Paratype. **Occurrence:** catalogNumber: BMNHE1722026; sex: Male; **Taxon:** scientificName: Lutzomyia (Helcocyrtomyia) monzonensis Ogusuku, Canales & Pérez, 1998; **Location:** country: Peru; stateProvince: Huánuco; municipality: Huamalíes; locality: Monzon, Paucacu; **Event:** eventDate: 05-22-97; eventRemarks: http://phlebotominaenhmtypes.myspecies.info/node/145; **Record Level:** institutionCode: NHMUK; basisOfRecord: Preserved Specimen

##### Distribution

Peru

##### Notes

Valid species in Lutzomyia (Helcocyrtomyia).

#### Pintomyia odax

Fairchild & Hertig, 1961

Phlebotomus odax Fairchild & Hertig, 1961 (Fairchild and Hertig 1961)

##### Materials

- **Type status:** Paratype. **Occurrence:** catalogNumber: BMNHE1722052; sex: Female; **Taxon:** scientificName: Pintomyia (Pifanomyia) odax (Fairchild & Hertig, 1961); **Location:** country: Honduras; stateProvince: Atlántida; municipality: Tela; locality: Lancetilla Valley, Tela; **Event:** eventDate: 06/01/54; eventRemarks: http://phlebotominaenhmtypes.myspecies.info/node/171; **Record Level:** institutionCode: NHMUK; basisOfRecord: Preserved Specimen
- **Type status:** Paratype. **Occurrence:** catalogNumber: BMNHE1722054; sex: Male; **Taxon:** scientificName: Pintomyia (Pifanomyia) odax (Fairchild & Hertig, 1961); **Location:** country: Panama; stateProvince: Bocas del Toro; locality: Finca Nievecita, Almirante; **Event:** eventDate: 17/06/50; eventRemarks: http://phlebotominaenhmtypes.myspecies.info/node/173; **Record Level:** institutionCode: NHMUK; basisOfRecord: Preserved Specimen
- **Type status:** Paratype. **Occurrence:** catalogNumber: BMNHE1722055; sex: Female; **Taxon:** scientificName: Pintomyia (Pifanomyia) odax (Fairchild & Hertig, 1961); **Location:** country: Honduras; stateProvince: Atlántida; municipality: Tela; locality: Lancetilla Valley, Tela; **Event:** eventDate: 23/12/53; eventRemarks: http://phlebotominaenhmtypes.myspecies.info/node/174; **Record Level:** institutionCode: NHMUK; basisOfRecord: Preserved Specimen

##### Distribution

Brazil, Costa Rica, French Guiana, Guatemala, Honduras, Nicaragua, Panama, Venezuela

##### Notes

Valid species in Pintomyia (Pifanomyia).

#### Oligodontomyia oligodonta

Young, Pérez & Romero, 1985

Lutzomyia oligodonta Young, Pérez & Romero, 1985 (Young et al. 1985)

##### Materials

- **Type status:** Paratype. **Occurrence:** catalogNumber: BMNHE1722098; sex: Male; **Taxon:** scientificName: *Oligodontomyia oligodonta* (Young, Pérez & Romero, 1985); **Location:** country: Peru; stateProvince: Lima; locality: Cocachacra; **Event:** eventDate: 01/05/63; eventRemarks: http://phlebotominaenhmtypes.myspecies.info/node/217; **Record Level:** institutionCode: NHMUK; basisOfRecord: Preserved Specimen

##### Distribution

Peru

##### Notes

Valid species in *Oligodontomyia*.

#### Bichromomyia olmeca novica

Young & Arias, 1982

Lutzomyia olmeca novica Young & Arias, 1982 (Young and Arias 1982)

##### Materials

- **Type status:** Paratype. **Occurrence:** catalogNumber: BMNHE1722066; sex: Female; **Taxon:** scientificName: *Bichromomyia olmeca novica* (Young & Arias, 1982); **Location:** country: Brazil; stateProvince: Amazonas; locality: 43km NE Manaus, at Campinas; **Event:** eventDate: 03-21-79; eventRemarks: http://phlebotominaenhmtypes.myspecies.info/node/185; **Record Level:** institutionCode: NHMUK; basisOfRecord: Preserved Specimen
- **Type status:** Paratype. **Occurrence:** catalogNumber: BMNHE1722067; sex: Male; **Taxon:** scientificName: *Bichromomyia olmeca novica* (Young & Arias, 1982); **Location:** country: Brazil; stateProvince: Amazonas; locality: 25km E of Manaus, Ducke Reserve; **Event:** eventDate: 16-19/03/1979; eventRemarks: http://phlebotominaenhmtypes.myspecies.info/node/186; **Record Level:** institutionCode: NHMUK; basisOfRecord: Preserved Specimen

##### Distribution

Brazil, Peru

##### Notes

Valid species in *Bichromomyia*.

#### Bichromomyia olmeca olmeca

Vargas & Díaz-Nájera, 1959

Phlebotomus olmecus Vargas & Díaz-Nájera, 1959 (Vargas and Díaz Nájera 1959)

##### Materials

- **Type status:** Allotype. **Occurrence:** catalogNumber: BMNHE1251319; sex: Female; **Taxon:** scientificName: *Bichromomyia olmeca olmeca* (Vargas & Díaz-Nájera, 1959); **Location:** country: Mexico; stateProvince: Tabasco; municipality: Teapa; locality: Sta A-0; **Event:** eventDate: 12-16-53; eventRemarks: http://phlebotominaenhmtypes.myspecies.info/node/91; **Record Level:** institutionCode: NHMUK; basisOfRecord: Preserved Specimen

##### Distribution

Belize, Costa Rica, Guatemala, Honduras, Mexico, Nicaragua

##### Notes

Valid species in *Bichromomyia*.

#### Pintomyia oresbia

(Fairchild & Hertig, 1961)

Phlebotomus oresbia Fairchild & Hertig, 1961 (Fairchild and Hertig 1961)

##### Materials

- **Type status:** Paratype. **Occurrence:** catalogNumber: BMNHE1722053; sex: Female; **Taxon:** scientificName: Pintomyia (Pifanomyia) oresbia (Fairchild & Hertig, 1961); **Location:** country: Panama; stateProvince: Chiriquí; municipality: Renacimiento; locality: Rio Candela, Chiriquí; **Event:** eventDate: 11/08/51; eventRemarks: http://phlebotominaenhmtypes.myspecies.info/node/172; **Record Level:** institutionCode: NHMUK; basisOfRecord: Preserved Specimen

##### Distribution

Costa Rica, Panama

##### Notes

Valid species in Pintomyia (Pifanomyia).

#### Lutzomyia peruensis

Shannon, 1929

Phlebotomus peruensis Shannon, 1929 (Shannon 1929)

##### Materials

- **Type status:** Syntype. **Occurrence:** catalogNumber: BMNHE1722139; sex: Male; **Taxon:** scientificName: Lutzomyia (Helcocyrtomyia) peruensis (Shannon, 1929); **Location:** country: Peru; stateProvince: Lima; locality: Matucana; **Event:** eventDate: 06-19-28; eventRemarks: http://phlebotominaenhmtypes.myspecies.info/node/258; **Record Level:** institutionCode: NHMUK; basisOfRecord: Preserved Specimen

##### Distribution

Bolivia, Peru

##### Notes

Valid species in Lutzomyia (Helcocyrtomyia).

#### Warileya phlebotomanica

Hertig, 1948

Warileya phlebotomanica Hertig, 1948 (Hertig 1948)

##### Materials

- **Type status:** Paratype. **Occurrence:** catalogNumber: BMNHE1722044; sex: Female; **Taxon:** scientificName: *Warileya phlebotomanica* Hertig, 1948; **Location:** country: Peru; stateProvince: Lima; municipality: Huarochiri; locality: Acequia cueva, Matucana; **Event:** eventDate: 08-25-45; eventRemarks: http://phlebotominaenhmtypes.myspecies.info/node/163; **Record Level:** institutionCode: NHMUK; basisOfRecord: Preserved Specimen
- **Type status:** Paratype. **Occurrence:** catalogNumber: BMNHE1722045; sex: Female; **Taxon:** scientificName: *Warileya phlebotomanica* Hertig, 1949; **Location:** country: Peru; stateProvince: Lima; municipality: Huarochiri; locality: Rio Rimac, Matucana; **Event:** month: 8; day: 23; eventRemarks: http://phlebotominaenhmtypes.myspecies.info/node/164; **Record Level:** institutionCode: NHMUK; basisOfRecord: Preserved Specimen

##### Distribution

Ecuador, Peru

##### Notes

Valid species in *Warileya*.

#### Pintomyia pia

Fairchild & Hertig, 1961

Phlebotomus pius Fairchild & Hertig, 1961 (Fairchild and Hertig 1961)

##### Materials

- **Type status:** Paratype. **Occurrence:** catalogNumber: BMNHE1722103; sex: Male; **Taxon:** scientificName: Pintomyia (Pifanomyia) pia (Fairchild & Hertig, 1961); **Location:** country: Panama; stateProvince: Chiriqui; locality: Lagunas del Volcán; **Event:** eventDate: 05-19-50; eventRemarks: http://phlebotominaenhmtypes.myspecies.info/node/222; **Record Level:** institutionCode: NHMUK; basisOfRecord: Preserved Specimen
- **Type status:** Paratype. **Occurrence:** catalogNumber: BMNHE1722104; sex: Male; **Taxon:** scientificName: Pintomyia (Pifanomyia) pia (Fairchild & Hertig, 1961); **Location:** country: Panama; stateProvince: Chiriqui; locality: Sta. Clara, El Volcan; **Event:** eventDate: 08-11-50; eventRemarks: http://phlebotominaenhmtypes.myspecies.info/node/223; **Record Level:** institutionCode: NHMUK; basisOfRecord: Preserved Specimen

##### Distribution

Bolivia, Colombia, Costa Rica, Panama, Peru, Venezuela

##### Notes

Valid species in Pintomyia (Pifanomyia).

#### Evandromyia pinottii

Damasceno & Arouck, 1956

Flebotomus pinottii Damasceno & Arouck, 1956 (Damasceno and Arouck 1956)

##### Materials

- **Type status:** Paratype. **Occurrence:** catalogNumber: BMNHE1721996; sex: Male; **Taxon:** scientificName: Evandromyia (Evandromyia) pinottii (Damasceno & Arouck, 1956); **Location:** country: Brazil; locality: Rio Acará; **Event:** eventRemarks: http://phlebotominaenhmtypes.myspecies.info/node/115; **Record Level:** institutionCode: NHMUK; basisOfRecord: Preserved Specimen

##### Distribution

Brazil, French Guiana, Venezuela

##### Notes

Valid species in Evandromyia (Evandromyia).

#### Pintomyia quasitownsendi

Osorno, Osorno-Mesa & Morales, 1972

Lutzomyia quasitownsendi Osorno, Osorno-Mesa & Morales, 1972 (Osorno et al. 1972)

##### Materials

- **Type status:** Paratype. **Occurrence:** catalogNumber: BMNHE1722092; sex: Male; **Taxon:** scientificName: Pintomyia (Pifanomyia) quasitownsendi (Osorno, Osorno-Mesa & Morales, 1972); **Location:** country: Colombia; stateProvince: Santander; municipality: Socorro; **Event:** eventDate: 12-09-67; eventRemarks: http://phlebotominaenhmtypes.myspecies.info/node/211; **Record Level:** institutionCode: NHMUK; basisOfRecord: Preserved Specimen
- **Type status:** Paratype. **Occurrence:** catalogNumber: BMNHE1722093; sex: Female; **Taxon:** scientificName: Pintomyia (Pifanomyia) quasitownsendi (Osorno, Osorno-Mesa & Morales, 1972); **Location:** country: Colombia; stateProvince: Santander; municipality: Barbosa; **Event:** eventDate: 10-30-68; eventRemarks: http://phlebotominaenhmtypes.myspecies.info/node/212; **Record Level:** institutionCode: NHMUK; basisOfRecord: Preserved Specimen
- **Type status:** Paratype. **Occurrence:** catalogNumber: BMNHE1722129; sex: Female; **Taxon:** scientificName: Pintomyia (Pifanomyia) quasitownsendi (Osorno, Osorno-Mesa & Morales, 1972); **Location:** country: Colombia; stateProvince: Santander; municipality: Barbosa; **Event:** eventDate: 10-30-68; eventRemarks: http://phlebotominaenhmtypes.myspecies.info/node/248; **Record Level:** institutionCode: NHMUK; basisOfRecord: Preserved Specimen
- **Type status:** Paratype. **Occurrence:** catalogNumber: BMNHE1722130; sex: Female; **Taxon:** scientificName: Pintomyia (Pifanomyia) quasitownsendi (Osorno, Osorno-Mesa & Morales, 1972); **Location:** country: Colombia; stateProvince: Santander; municipality: Güepsa; **Event:** eventDate: 06-22-67; eventRemarks: http://phlebotominaenhmtypes.myspecies.info/node/249; **Record Level:** institutionCode: NHMUK; basisOfRecord: Preserved Specimen

##### Distribution

Colombia

##### Notes

Valid species in Pintomyia (Pifanomyia).

#### Nyssomyia richardwardi

Ready & Fraiha, 1981

Lutzomyia richardwardi Ready & Fraiha, 1981 (Ready and Fraiha 1981)

##### Materials

- **Type status:** Paratype. **Occurrence:** catalogNumber: BMNHE1251320; sex: Female; **Taxon:** scientificName: *Nyssomyia richardwardi* (Ready & Fraiha, 1981); **Location:** country: Brazil; stateProvince: Pará; locality: Altamira-Itaituba rd km 43; **Event:** eventDate: 08-21-71; eventRemarks: http://phlebotominaenhmtypes.myspecies.info/node/92; **Record Level:** institutionCode: NHMUK; basisOfRecord: Preserved Specimen
- **Type status:** Paratype. **Occurrence:** catalogNumber: BMNHE1722063; sex: Female; **Taxon:** scientificName: *Nyssomyia richardwardi* (Ready & Fraiha, 1981); **Location:** country: Brazil; stateProvince: Pará; locality: km 43 Altamira to Itaituba rd; **Event:** eventDate: 08-21-71; eventRemarks: http://phlebotominaenhmtypes.myspecies.info/node/182; **Record Level:** institutionCode: NHMUK; basisOfRecord: Preserved Specimen
- **Type status:** Paratype. **Occurrence:** catalogNumber: BMNHE1722064; sex: Female; **Taxon:** scientificName: *Nyssomyia richardwardi* (Ready & Fraiha, 1981); **Location:** country: Brazil; stateProvince: Pará; locality: km 43 Altamira to Itaituba rd; **Event:** eventDate: 08-21-71; eventRemarks: http://phlebotominaenhmtypes.myspecies.info/node/183; **Record Level:** institutionCode: NHMUK; basisOfRecord: Preserved Specimen

##### Distribution

Bolivia, Brazil, Colombia, Ecuador, Peru

##### Notes

Valid species in *Nyssomyia*.

#### Dampfomyia rosabali

Fairchild & Hertig, 1956

Phlebotomus rosabali Fairchild & Hertig, 1956 (Fairchild and Hertig 1956)

##### Materials

- **Type status:** Paratype. **Occurrence:** catalogNumber: BMNHE1721991; sex: Female; **Taxon:** scientificName: Dampfomyia (Dampfomyia) rosabali (Fairchild & Hertig, 1956); **Location:** country: Costa Rica; stateProvince: Puntarenas; locality: Barranca, Finca Socorrito; **Event:** eventDate: 12-17-51; eventRemarks: http://phlebotominaenhmtypes.myspecies.info/node/110; **Record Level:** institutionCode: NHMUK; basisOfRecord: Preserved Specimen
- **Type status:** Paratype. **Occurrence:** catalogNumber: BMNHE1722048; sex: Male; **Taxon:** scientificName: Dampfomyia (Dampfomyia) rosabali (Fairchild & Hertig, 1956); **Location:** country: Colombia; stateProvince: Cauca; municipality: Bolívar; locality: Bolívar (Cauca); **Event:** eventDate: 23/01/44; eventRemarks: http://phlebotominaenhmtypes.myspecies.info/node/167; **Record Level:** institutionCode: NHMUK; basisOfRecord: Preserved Specimen

##### Distribution

Colombia, Costa Rica, Panama

##### Notes

Valid species in Dampfomyia (Dampfomyia).

#### Trichophoromyia rostrans

Summers, 1912

Phlebotomus rostrans Summers, 1912 (Summers 1912)

##### Materials

- **Type status:** Paralectotype. **Occurrence:** catalogNumber: BMNHE1722082; sex: Male; **Taxon:** scientificName: *Trichophoromyia rostrans* (Summers, 1912); **Location:** locality: Rio Javari, Peru/Brazil border; **Event:** year: 1912; eventRemarks: http://phlebotominaenhmtypes.myspecies.info/node/201; **Record Level:** institutionCode: NHMUK; basisOfRecord: Preserved Specimen
- **Type status:** Paralectotype. **Occurrence:** catalogNumber: BMNHE1722083; sex: Female; **Taxon:** scientificName: *Trichophoromyia rostrans* (Summers, 1912); **Location:** locality: Rio Javari, Peru/Brazil border; **Event:** year: 1912; eventRemarks: http://phlebotominaenhmtypes.myspecies.info/node/202; **Record Level:** institutionCode: NHMUK; basisOfRecord: Preserved Specimen
- **Type status:** Paralectotype. **Occurrence:** catalogNumber: BMNHE1722124; sex: Female; **Taxon:** scientificName: *Trichophoromyia rostrans* (Summers, 1912); **Location:** locality: Rio Javari, Peru/Brazil border; **Event:** year: 1912; eventRemarks: http://phlebotominaenhmtypes.myspecies.info/node/243; **Record Level:** institutionCode: NHMUK; basisOfRecord: Preserved Specimen
- **Type status:** Lectotype. **Occurrence:** catalogNumber: BMNHE1722125; sex: Female; **Taxon:** scientificName: *Trichophoromyia rostrans* (Summers, 1912); **Location:** locality: Rio Javari, Peru/Brazil border; **Event:** eventRemarks: http://phlebotominaenhmtypes.myspecies.info/node/244; **Record Level:** institutionCode: NHMUK; basisOfRecord: Preserved Specimen

##### Distribution

Brazil

##### Notes

Valid species in *Trichophoromyia*. Lectotype designated by Lewis 1967b.

#### Warileya rotundipennis

Fairchild & Hertig, 1951

Warileya rotundipennis Fairchild & Hertig, 1951 (Fairchild and Hertig 1951b)

##### Materials

- **Type status:** Paratype. **Occurrence:** catalogNumber: BMNHE1721982; sex: Female; **Taxon:** scientificName: *Warileya rotundipennis* Fairchild & Hertig, 1951; **Location:** country: Panama; locality: Cerro Campana; **Event:** eventDate: 08-24-50; eventRemarks: http://phlebotominaenhmtypes.myspecies.info/node/101; **Record Level:** institutionCode: NHMUK; basisOfRecord: Preserved Specimen
- **Type status:** Paratype. **Occurrence:** catalogNumber: BMNHE1721983; sex: Male; **Taxon:** scientificName: *Warileya rotundipennis* Fairchild & Hertig, 1951; **Location:** country: Panama; locality: Cerro Campana; **Event:** eventDate: 08-24-50; eventRemarks: http://phlebotominaenhmtypes.myspecies.info/node/102; **Record Level:** institutionCode: NHMUK; basisOfRecord: Preserved Specimen
- **Type status:** Paratype. **Occurrence:** catalogNumber: BMNHE1722046; sex: Female; **Taxon:** scientificName: *Warileya rotundipennis* Fairchild & Hertig, 1953; **Location:** country: Panama; locality: Cerro Campana; **Event:** eventDate: 08-24-50; eventRemarks: http://phlebotominaenhmtypes.myspecies.info/node/165; **Record Level:** institutionCode: NHMUK; basisOfRecord: Preserved Specimen

##### Distribution

Bolivia, Colombia, Costa Rica, Panama, Peru

##### Notes

Valid species in *Warileya*.

#### Evandromyia sallesi

Galvão & Coutinho, 1939

Flebotomus sallesi Galvão & Coutinho, 1939 (Galvão and Coutinho 1939)

##### Materials

- **Type status:** Paratype. **Occurrence:** catalogNumber: BMNHE1722069; sex: Female; **Taxon:** scientificName: Evandromyia (Barrettomyia) sallesi (Galvão & Coutinho, 1939); **Location:** country: Brazil; stateProvince: São Paulo; municipality: Araçatuba; **Event:** year: 1935; eventRemarks: http://phlebotominaenhmtypes.myspecies.info/node/188; **Record Level:** institutionCode: NHMUK; basisOfRecord: Preserved Specimen

##### Distribution

Argentina, Bolivia, Brazil, Ecuador, Paraguay, Peru

##### Notes

Valid species in Evandromyia (Barrettomyia).

#### Lutzomyia scorzai

Ortiz, 1965

Phlebotomus scorzai Ortiz, 1965 (Ortíz 1965)

##### Materials

- **Type status:** Paratype. **Occurrence:** catalogNumber: BMNHE1722072; sex: Male; **Taxon:** scientificName: Lutzomyia (Helcocyrtomyia) scorzai (Ortiz, 1965); **Location:** country: Venezuela; locality: Rancho Grande; **Event:** year: 1965; eventRemarks: http://phlebotominaenhmtypes.myspecies.info/node/191; **Record Level:** institutionCode: NHMUK; basisOfRecord: Preserved Specimen

##### Distribution

Colombia, Peru, Venezuela

##### Notes

Valid species in Lutzomyia (Helcocyrtomyia).

#### Nyssomyia shawi

Fraiha, Ward & Ready, 1981

Lutzomyia shawi Fraiha, Ward & Ready, 1981 (Fraiha et al. 1981)

##### Materials

- **Type status:** Paratype. **Occurrence:** catalogNumber: BMNHE1251316; sex: Male; **Taxon:** scientificName: *Nyssomyia shawi* (Fraiha, Ward & Ready, 1981); **Location:** country: Brazil; stateProvince: Pará; municipality: Marabá; locality: Serra dos Carajás; **Event:** year: 1975; month: 2; eventRemarks: http://phlebotominaenhmtypes.myspecies.info/node/88; **Record Level:** institutionCode: NHMUK; basisOfRecord: Preserved Specimen
- **Type status:** Paratype. **Occurrence:** catalogNumber: BMNHE1251317; sex: Female; **Taxon:** scientificName: *Nyssomyia shawi* (Fraiha, Ward & Ready, 1981); **Location:** country: Brazil; stateProvince: Pará; municipality: Marabá; locality: Serra dos Carajás; **Event:** eventDate: 09-22-74; eventRemarks: http://phlebotominaenhmtypes.myspecies.info/node/89; **Record Level:** institutionCode: NHMUK; basisOfRecord: Preserved Specimen
- **Type status:** Paratype. **Occurrence:** catalogNumber: BMNHE1722061; sex: Male; **Taxon:** scientificName: *Nyssomyia shawi* (Fraiha, Ward & Ready, 1981); **Location:** country: Brazil; stateProvince: Pará; municipality: Marabá; locality: Serra dos Carajás; **Event:** year: 1975; month: 2; eventRemarks: http://phlebotominaenhmtypes.myspecies.info/node/180; **Record Level:** institutionCode: NHMUK; basisOfRecord: Preserved Specimen
- **Type status:** Paratype. **Occurrence:** catalogNumber: BMNHE1722062; sex: Female; **Taxon:** scientificName: *Nyssomyia shawi* (Fraiha, Ward & Ready, 1981); **Location:** country: Brazil; stateProvince: Pará; municipality: Marabá; locality: Serra dos Carajás; **Event:** eventDate: 09-23-74; eventRemarks: http://phlebotominaenhmtypes.myspecies.info/node/181; **Record Level:** institutionCode: NHMUK; basisOfRecord: Preserved Specimen
- **Type status:** Paratype. **Occurrence:** catalogNumber: BMNHE1722065; sex: Female; **Taxon:** scientificName: *Nyssomyia shawi* (Fraiha, Ward & Ready, 1981); **Location:** country: Brazil; stateProvince: Pará; municipality: Marabá; locality: Serra dos Carajás; **Event:** eventDate: 09-23-74; eventRemarks: http://phlebotominaenhmtypes.myspecies.info/node/184; **Record Level:** institutionCode: NHMUK; basisOfRecord: Preserved Specimen

##### Distribution

Bolivia, Brazil, Colombia, Peru

##### Notes

Valid species in *Nyssomyia*.

#### Pintomyia spinicrassa

Morales, Osorno-Mesa, Osorno & Hoyos, 1969

Lutzomyia spinicrassa Morales, Osorno-Mesa, Osorno & Hoyos, 1969 (Morales et al. 1969)

##### Materials

- **Type status:** Paratype. **Occurrence:** catalogNumber: BMNHE1722094; sex: Female; **Taxon:** scientificName: Pintomyia (Pifanomyia) spinicrassa (Morales, Osorno-Mesa, Osorno & Hoyos, 1969); **Location:** country: Colombia; stateProvince: Boyacá; locality: Guayatá - Chitavita; **Event:** eventDate: 08-09-68; eventRemarks: http://phlebotominaenhmtypes.myspecies.info/node/213; **Record Level:** institutionCode: NHMUK; basisOfRecord: Preserved Specimen
- **Type status:** Paratype. **Occurrence:** catalogNumber: BMNHE1722095; sex: Male; **Taxon:** scientificName: Pintomyia (Pifanomyia) spinicrassa (Morales, Osorno-Mesa, Osorno & Hoyos, 1969); **Location:** country: Colombia; stateProvince: Boyacá; locality: Guayatá - Chitavita; **Event:** eventDate: 08-09-68; eventRemarks: http://phlebotominaenhmtypes.myspecies.info/node/214; **Record Level:** institutionCode: NHMUK; basisOfRecord: Preserved Specimen
- **Type status:** Paratype. **Occurrence:** catalogNumber: BMNHE1722131; sex: Female; **Taxon:** scientificName: Pintomyia (Pifanomyia) spinicrassa (Morales, Osorno-Mesa, Osorno & Hoyos, 1969); **Location:** country: Colombia; stateProvince: Boyacá; locality: Guayatá - Chitavita; **Event:** eventDate: 08-09-68; eventRemarks: http://phlebotominaenhmtypes.myspecies.info/node/250; **Record Level:** institutionCode: NHMUK; basisOfRecord: Preserved Specimen
- **Type status:** Paratype. **Occurrence:** catalogNumber: BMNHE1722132; sex: Female; **Taxon:** scientificName: Pintomyia (Pifanomyia) spinicrassa (Morales, Osorno-Mesa, Osorno & Hoyos, 1969); **Location:** country: Colombia; stateProvince: Boyacá; locality: Guayatá - Chitavita; **Event:** eventDate: 08-09-68; eventRemarks: http://phlebotominaenhmtypes.myspecies.info/node/251; **Record Level:** institutionCode: NHMUK; basisOfRecord: Preserved Specimen

##### Distribution

Colombia, Venezuela

##### Notes

Valid species in Pintomyia (Pifanomyia).

#### Lutzomyia strictivilla

Young, 1979

Lutzomyia strictivilla Young, 1979 (Young 1979)

##### Materials

- **Type status:** Paratype. **Occurrence:** catalogNumber: BMNHE1722009; sex: Male; **Taxon:** scientificName: Lutzomyia (Helcocyrtomyia) strictivilla Young, 1979; **Location:** country: Colombia; stateProvince: Antioquia; municipality: Zaragoza; locality: 24km SW of Zaragoza at Rio Anori; **Event:** eventDate: 04-14-71; eventRemarks: http://phlebotominaenhmtypes.myspecies.info/node/128; **Record Level:** institutionCode: NHMUK; basisOfRecord: Preserved Specimen

##### Distribution

Colombia, Ecuador, Venezuela

##### Notes

Valid species in Lutzomyia (Helcocyrtomyia).

#### Nyssomyia trapidoi

Fairchild & Hertig, 1952

Phlebotomus trapidoi Fairchild & Hertig, 1952 (Fairchild and Hertig 1952)

##### Materials

- **Type status:** Paratype. **Occurrence:** catalogNumber: BMNHE1251314; sex: Male; **Taxon:** scientificName: *Nyssomyia trapidoi* (Fairchild & Hertig, 1952); **Location:** country: Panama; stateProvince: Bocas del Toro; locality: Almirante, Finca Nievecita; **Event:** eventDate: 06-21-50; eventRemarks: http://phlebotominaenhmtypes.myspecies.info/node/86; **Record Level:** institutionCode: NHMUK
- **Type status:** Paratype. **Occurrence:** catalogNumber: BMNHE1722060; **Taxon:** scientificName: *Nyssomyia trapidoi* (Fairchild & Hertig, 1952); **Location:** country: Panama; stateProvince: Bocas del Toro; locality: Almirante; **Event:** eventDate: 22/07/50; eventRemarks: http://phlebotominaenhmtypes.myspecies.info/node/179; **Record Level:** institutionCode: NHMUK; basisOfRecord: Preserved Specimen

##### Distribution

Colombia, Costa Rica, Ecuador, Guatemala, Hounduras, Nicaragua, Panama

##### Notes

Valid species in *Nyssomyia*.

#### Micropygomyia trinidadensis

Newstead, 1922

Phlebotomus trinidadensis Newstead, 1922 (Newstead 1922)

##### Materials

- **Type status:** Syntype. **Occurrence:** catalogNumber: BMNHE1721999; sex: Male; **Taxon:** scientificName: Micropygomyia (Sauromyia) trinidadensis (Newstead, 1922); **Location:** country: Trinidad and Tabago; **Event:** eventDate: 01-07-22; eventRemarks: http://phlebotominaenhmtypes.myspecies.info/node/118; **Record Level:** institutionCode: NHMUK; basisOfRecord: Preserved Specimen
- **Type status:** Syntype. **Occurrence:** catalogNumber: BMNHE1722000; sex: Female; **Taxon:** scientificName: Micropygomyia (Sauromyia) trinidadensis (Newstead, 1922); **Location:** country: Trinidad and Tabago; **Event:** eventDate: 01-07-22; eventRemarks: http://phlebotominaenhmtypes.myspecies.info/node/119; **Record Level:** institutionCode: NHMUK; basisOfRecord: Preserved Specimen
- **Type status:** Syntype. **Occurrence:** catalogNumber: BMNHE1722001; sex: Female; **Taxon:** scientificName: Micropygomyia (Sauromyia) trinidadensis (Newstead, 1922); **Location:** country: Trinidad and Tabago; **Event:** eventDate: 01-07-22; eventRemarks: http://phlebotominaenhmtypes.myspecies.info/node/120; **Record Level:** institutionCode: NHMUK; basisOfRecord: Preserved Specimen
- **Type status:** Syntype. **Occurrence:** catalogNumber: BMNHE1722002; sex: Male; **Taxon:** scientificName: Micropygomyia (Sauromyia) trinidadensis (Newstead, 1922); **Location:** country: Trinidad and Tabago; **Event:** eventDate: 01-07-22; eventRemarks: http://phlebotominaenhmtypes.myspecies.info/node/121; **Record Level:** institutionCode: NHMUK; basisOfRecord: Preserved Specimen
- **Type status:** Syntype. **Occurrence:** catalogNumber: BMNHE1722003; sex: Male; **Taxon:** scientificName: Micropygomyia (Sauromyia) trinidadensis (Newstead, 1922); **Location:** country: Trinidad and Tabago; **Event:** eventDate: 01-07-22; eventRemarks: http://phlebotominaenhmtypes.myspecies.info/node/122; **Record Level:** institutionCode: NHMUK; basisOfRecord: Preserved Specimen
- **Type status:** Syntype. **Occurrence:** catalogNumber: BMNHE1722004; sex: Female; **Taxon:** scientificName: Micropygomyia (Sauromyia) trinidadensis (Newstead, 1922); **Location:** country: Trinidad and Tabago; **Event:** eventDate: 01-07-22; eventRemarks: http://phlebotominaenhmtypes.myspecies.info/node/123; **Record Level:** institutionCode: NHMUK; basisOfRecord: Preserved Specimen
- **Type status:** Syntype. **Occurrence:** catalogNumber: BMNHE1722005; sex: Female; **Taxon:** scientificName: Micropygomyia (Sauromyia) trinidadensis (Newstead, 1922); **Location:** country: Trinidad and Tabago; **Event:** eventDate: 01-07-22; eventRemarks: http://phlebotominaenhmtypes.myspecies.info/node/124; **Record Level:** institutionCode: NHMUK; basisOfRecord: Preserved Specimen
- **Type status:** Syntype. **Occurrence:** catalogNumber: BMNHE1722006; sex: Female; **Taxon:** scientificName: Micropygomyia (Sauromyia) trinidadensis (Newstead, 1922); **Location:** country: Trinidad and Tabago; **Event:** eventDate: 01-07-22; eventRemarks: http://phlebotominaenhmtypes.myspecies.info/node/125; **Record Level:** institutionCode: NHMUK; basisOfRecord: Preserved Specimen

##### Distribution

Belize, Bolivia, Brazil, Colombia, Costa Rica, Ecuador, El Salvador, French Guiana, Guatemala, Honduras, Mexico, Nicaragua, Peru, Panama, Surinam, Trinidad and Tobago, Venezuela

##### Notes

Valid species in Micropygomyia (Sauromyia).

#### Trichopygomyia triramula

Fairchild & Hertig, 1952

Phlebotomus triramulus Fairchild & Hertig, 1952 (Fairchild and Hertig 1952)

##### Materials

- **Type status:** Paratype. **Occurrence:** catalogNumber: BMNHE1722086; sex: Female; **Taxon:** scientificName: *Trichopygomyia triramula* (Fairchild & Hertig, 1952); **Location:** country: Panama; stateProvince: Panamá; locality: Cerro Jefe, La Victoria; **Event:** eventDate: 08-29-50; eventRemarks: http://phlebotominaenhmtypes.myspecies.info/node/205; **Record Level:** institutionCode: NHMUK; basisOfRecord: Preserved Specimen
- **Type status:** Paratype. **Occurrence:** catalogNumber: BMNHE1722087; sex: Male; **Taxon:** scientificName: *Trichopygomyia triramula* (Fairchild & Hertig, 1952); **Location:** country: Panama; stateProvince: Colón; locality: Palenque; **Event:** eventDate: 09-15-49; eventRemarks: http://phlebotominaenhmtypes.myspecies.info/node/206; **Record Level:** institutionCode: NHMUK; basisOfRecord: Preserved Specimen

##### Distribution

Belize, Colombia, Costa Rica, Ecuador, Guatemala, Mexico, Panama

##### Notes

Valid species in *Trichopygomyia*.

#### Trichophoromyia ubiquitalis

Mangabeira, 1942

Flebotomus ubiquitalis Mangabeira, 1942 (Mangabeira 1942)

##### Materials

- **Type status:** Syntype. **Occurrence:** catalogNumber: BMNHE1722084; sex: Male; **Taxon:** scientificName: *Trichophoromyia ubiquitalis* (Mangabeira, 1942); **Location:** country: Brazil; stateProvince: Pará; municipality: Belém; locality: Aura; **Event:** eventDate: 10-08-40; eventRemarks: http://phlebotominaenhmtypes.myspecies.info/node/203; **Record Level:** institutionCode: NHMUK; basisOfRecord: Preserved Specimen
- **Type status:** Syntype. **Occurrence:** catalogNumber: BMNHE1722085; sex: Male; **Taxon:** scientificName: *Trichophoromyia ubiquitalis* (Mangabeira, 1942); **Location:** country: Brazil; stateProvince: Pará; municipality: Belém; locality: Aura; **Event:** eventDate: 10-08-40; eventRemarks: http://phlebotominaenhmtypes.myspecies.info/node/204; **Record Level:** institutionCode: NHMUK; basisOfRecord: Preserved Specimen
- **Type status:** Syntype. **Occurrence:** catalogNumber: BMNHE1722126; sex: Male; **Taxon:** scientificName: *Trichophoromyia ubiquitalis* (Mangabeira, 1942); **Location:** country: Brazil; stateProvince: Pará; municipality: Belém; locality: Aura; **Event:** eventDate: 10-08-40; eventRemarks: http://phlebotominaenhmtypes.myspecies.info/node/245; **Record Level:** institutionCode: NHMUK; basisOfRecord: Preserved Specimen
- **Type status:** Syntype. **Occurrence:** catalogNumber: BMNHE1722127; sex: Male; **Taxon:** scientificName: *Trichophoromyia ubiquitalis* (Mangabeira, 1942); **Location:** country: Brazil; stateProvince: Pará; municipality: Belém; locality: Aura; **Event:** eventDate: 10-08-40; eventRemarks: http://phlebotominaenhmtypes.myspecies.info/node/246; **Record Level:** institutionCode: NHMUK; basisOfRecord: Preserved Specimen
- **Type status:** Syntype. **Occurrence:** catalogNumber: BMNHE1722128; sex: Male; **Taxon:** scientificName: *Trichophoromyia ubiquitalis* (Mangabeira, 1942); **Location:** country: Brazil; stateProvince: Pará; municipality: Belém; locality: Aura; **Event:** eventDate: 10-08-40; eventRemarks: http://phlebotominaenhmtypes.myspecies.info/node/247; **Record Level:** institutionCode: NHMUK; basisOfRecord: Preserved Specimen
- **Type status:** Syntype. **Occurrence:** catalogNumber: BMNHE1722140; sex: Male; **Taxon:** scientificName: *Trichophoromyia ubiquitalis* (Mangabeira, 1942); **Location:** country: Brazil; stateProvince: Pará; municipality: Belém; locality: Aura; **Event:** eventDate: 10-08-40; eventRemarks: http://phlebotominaenhmtypes.myspecies.info/node/259; **Record Level:** institutionCode: NHMUK; basisOfRecord: Preserved Specimen

##### Distribution

Bolivia, Brazil, Colombia, Ecuador, French Guiana, Peru, Surinam, Venezuela

##### Notes

Valid species in *Trichophoromyia*.

#### Nyssomyia umbratilis

Ward & Fraiha, 1977

Lutzomyia umbratilis Ward & Fraiha, 1977 (Ward and Fraiha 1977)

##### Materials

- **Type status:** Paratype. **Occurrence:** catalogNumber: BMNHE1722058; sex: Female; **Taxon:** scientificName: *Nyssomyia umbratilis* (Ward & Fraiha, 1977); **Location:** country: Brazil; stateProvince: Pará; locality: Rio Jari, Monte Dourado; **Event:** eventDate: 06/02/76; eventRemarks: http://phlebotominaenhmtypes.myspecies.info/node/177; **Record Level:** institutionCode: NHMUK; basisOfRecord: Preserved Specimen
- **Type status:** Paratype. **Occurrence:** catalogNumber: BMNHE1722059; sex: Female; **Taxon:** scientificName: *Nyssomyia umbratilis* (Ward & Fraiha, 1977); **Location:** country: Brazil; stateProvince: Pará; locality: Rio Jari, Monte Dourado; **Event:** eventDate: 12/11/76; eventRemarks: http://phlebotominaenhmtypes.myspecies.info/node/178; **Record Level:** institutionCode: NHMUK; basisOfRecord: Preserved Specimen

##### Distribution

Bolivia, Brazil, Colombia, French Guiana, Peru, Surinam, Venezuela

##### Notes

Valid species in *Nyssomyia*.

#### Lutzomyia vexillarius

Fairchild & Hertig, 1952

Phlebotomus vexillarius Fairchild & Hertig, 1952 (Fairchild and Hertig 1952)

##### Materials

- **Type status:** Paratype. **Occurrence:** catalogNumber: BMNHE1722027; sex: Male; **Taxon:** scientificName: Lutzomyia (Lutzomyia) vexillarius (Fairchild & Hertig, 1952); acceptedNameUsage: Lutzomyia (Lutzomyia) lichyi (Floch & Abonnenc, 1950); **Location:** country: Panama; stateProvince: Panamá; locality: Serrania majé, Cerro Chucanti; **Event:** eventDate: 03-05-50; eventRemarks: http://phlebotominaenhmtypes.myspecies.info/node/146; **Record Level:** institutionCode: NHMUK; basisOfRecord: Preserved Specimen

##### Distribution

Brazil, Colombia, Costa Rica, Ecuador, French Guiana, Panama, Peru, Trinidad and Tobago, Venezuela

##### Notes

Synonym of Lutzomyia (Lutzomyia) lichyi (Floch & Abonnenc, 1950) Floch and Abonnenc 1950. Synonymised by Floch and Kramer 1965.

#### Dampfomyia viriosa

Fairchild & Hertig, 1958

Phlebotomus viriosa Fairchild & Hertig, 1958 (Fairchild and Hertig 1958a)

##### Materials

- **Type status:** Paratype. **Occurrence:** catalogNumber: BMNHE1722020; sex: Female; **Taxon:** scientificName: Dampfomyia (Coromyia) viriosa (Fairchild & Hertig, 1958); **Location:** country: Panama; stateProvince: Bocas del Toro; locality: near camp No.1, Almirante; **Event:** eventDate: 05-17-51; eventRemarks: http://phlebotominaenhmtypes.myspecies.info/node/139; **Record Level:** institutionCode: NHMUK; basisOfRecord: Preserved Specimen
- **Type status:** Paratype. **Occurrence:** catalogNumber: BMNHE1722021; sex: Male; **Taxon:** scientificName: Dampfomyia (Coromyia) viriosa (Fairchild & Hertig, 1958); **Location:** country: Panama; stateProvince: Bocas del Toro; locality: Almirante; **Event:** eventDate: 03-24-52; eventRemarks: http://phlebotominaenhmtypes.myspecies.info/node/140; **Record Level:** institutionCode: NHMUK; basisOfRecord: Preserved Specimen
- **Type status:** Paratype. **Occurrence:** catalogNumber: BMNHE1722022; sex: Female; **Taxon:** scientificName: Dampfomyia (Coromyia) viriosa (Fairchild & Hertig, 1958); **Location:** country: Panama; stateProvince: Bocas del Toro; locality: Almirante; **Event:** eventDate: 09-17-52; eventRemarks: http://phlebotominaenhmtypes.myspecies.info/node/141; **Record Level:** institutionCode: NHMUK; basisOfRecord: Preserved Specimen
- **Type status:** Paratype. **Occurrence:** catalogNumber: BMNHE1722023; sex: Male; **Taxon:** scientificName: Dampfomyia (Coromyia) viriosa (Fairchild & Hertig, 1958); **Location:** country: Panama; stateProvince: Bocas del Toro; locality: near camp No.1, Almirante; **Event:** eventDate: 05-17-51; eventRemarks: http://phlebotominaenhmtypes.myspecies.info/node/142; **Record Level:** institutionCode: NHMUK; basisOfRecord: Preserved Specimen
- **Type status:** Paratype. **Occurrence:** catalogNumber: BMNHE1722024; sex: Female; **Taxon:** scientificName: Dampfomyia (Coromyia) viriosa (Fairchild & Hertig, 1958); **Location:** country: Panama; stateProvince: Bocas del Toro; locality: Almirante; **Event:** eventDate: 09-09-52; eventRemarks: http://phlebotominaenhmtypes.myspecies.info/node/143; **Record Level:** institutionCode: NHMUK; basisOfRecord: Preserved Specimen
- **Type status:** Paratype. **Occurrence:** catalogNumber: BMNHE1722025; sex: Male; **Taxon:** scientificName: Dampfomyia (Coromyia) viriosa (Fairchild & Hertig, 1958); **Location:** country: Panama; stateProvince: Bocas del Toro; locality: near camp No.1, Almirante; **Event:** eventDate: 05-17-51; eventRemarks: http://phlebotominaenhmtypes.myspecies.info/node/144; **Record Level:** institutionCode: NHMUK; basisOfRecord: Preserved Specimen

##### Distribution

Costa Rica, Panama

##### Notes

Valid species in Dampfomyia (Coromyia).

#### Evandromyia walkeri

Newstead, 1914

Phlebotomus walkeri Newstead 1914 (Newstead 1914)
Phlebotomus gasti Sherlock, 1962

##### Materials

- **Type status:** Lectotype. **Occurrence:** catalogNumber: BMNHE1722099; sex: Male; **Taxon:** scientificName: Evandromyia (Aldamyia) walkeri (Newstead, 1914); **Location:** locality: Rio Abuna, Brazil/Boliva border; **Event:** eventRemarks: http://phlebotominaenhmtypes.myspecies.info/node/218; **Record Level:** institutionCode: NHMUK; basisOfRecord: Preserved Specimen
- **Type status:** Paralectotype. **Occurrence:** catalogNumber: BMNHE1722100; sex: Male; **Taxon:** scientificName: Evandromyia (Aldamyia) walkeri (Newstead, 1914); **Location:** locality: Rio Abuna, Brazil/Boliva border; **Event:** eventRemarks: http://phlebotominaenhmtypes.myspecies.info/node/219; **Record Level:** institutionCode: NHMUK; basisOfRecord: Preserved Specimen
- **Type status:** Paralectotype. **Occurrence:** catalogNumber: BMNHE1722101; sex: Female; **Taxon:** scientificName: Evandromyia (Aldamyia) walkeri (Newstead, 1914); **Location:** locality: Rio Abuna, Brazil/Boliva border; **Event:** eventRemarks: http://phlebotominaenhmtypes.myspecies.info/node/220; **Record Level:** institutionCode: NHMUK; basisOfRecord: Preserved Specimen
- **Type status:** Paralectotype. **Occurrence:** catalogNumber: BMNHE1722102; sex: Female; **Taxon:** scientificName: Evandromyia (Aldamyia) walkeri (Newstead, 1914); **Location:** locality: Rio Abuna, Brazil/Boliva border; **Event:** eventRemarks: http://phlebotominaenhmtypes.myspecies.info/node/221; **Record Level:** institutionCode: NHMUK; basisOfRecord: Preserved Specimen
- **Type status:** Paralectotype. **Occurrence:** catalogNumber: BMNHE1722133; sex: Male; **Taxon:** scientificName: Evandromyia (Aldamyia) walkeri (Newstead, 1914); **Location:** country: Brazil; stateProvince: State of Rondonia; locality: Rio Abuna, Brazil/Boliva border; **Event:** eventRemarks: http://phlebotominaenhmtypes.myspecies.info/node/252; **Record Level:** institutionCode: NHMUK; basisOfRecord: Preserved Specimen
- **Type status:** Paralectotype. **Occurrence:** catalogNumber: BMNHE1722135; sex: Male; **Taxon:** scientificName: Evandromyia (Aldamyia) walkeri (Newstead, 1914); **Location:** country: Brazil; stateProvince: State of Rondonia; locality: Rio Abuna, Brazil/Boliva border; **Event:** eventRemarks: http://phlebotominaenhmtypes.myspecies.info/node/254; **Record Level:** institutionCode: NHMUK; basisOfRecord: Preserved Specimen
- **Type status:** Paralectotype. **Occurrence:** catalogNumber: BMNHE1722136; sex: Male; **Taxon:** scientificName: Evandromyia (Aldamyia) walkeri (Newstead, 1914); **Location:** country: Brazil; stateProvince: State of Rondonia; locality: Rio Abuna, Brazil/Boliva border; **Event:** eventRemarks: http://phlebotominaenhmtypes.myspecies.info/node/255; **Record Level:** institutionCode: NHMUK; basisOfRecord: Preserved Specimen
- **Type status:** Paralectotype. **Occurrence:** catalogNumber: BMNHE1722137; sex: Male; **Taxon:** scientificName: Evandromyia (Aldamyia) walkeri (Newstead, 1914); **Location:** country: Brazil; stateProvince: State of Rondonia; locality: Rio Abuna, Brazil/Boliva border; **Event:** eventRemarks: http://phlebotominaenhmtypes.myspecies.info/node/256; **Record Level:** institutionCode: NHMUK; basisOfRecord: Preserved Specimen

##### Distribution

Bolivia, Brazil, Colombia, Ecuador, French Guiana, Panama, Peru, Trinidad and Tobago, Venezuela

##### Notes

Valid species in Evandromyia (Aldamyia). Lectotype designated by Lewis 1967b.

#### Pintomyia xerophila

Young, Brener & Wargo, 1983

Lutzomyia xerophila Young, Brener & Wargo, 1983 (Young et al. 1983)

##### Materials

- **Type status:** Paratype. **Occurrence:** catalogNumber: BMNHE1722105; sex: Female; **Taxon:** scientificName: *Pintomyia xerophila* (Young, Brener & Wargo, 1983); **Location:** country: USA; stateProvince: California; municipality: Riverside; locality: Cahuilla Hills, Palm desert; **Event:** eventDate: 09-16-81; eventRemarks: http://phlebotominaenhmtypes.myspecies.info/node/224; **Record Level:** institutionCode: NHMUK; basisOfRecord: Preserved Specimen

##### Distribution

United States of America

##### Notes

Valid species in *Pintomyia (incertae sedis)*.

#### Nyssomyia ylephiletor

Fairchild & Hertig, 1952

Phlebotomus ylephiletor Fairchild & Hertig, 1952 (Fairchild and Hertig 1952)

##### Materials

- **Type status:** Paratype. **Occurrence:** catalogNumber: BMNHE1251315; sex: Male; **Taxon:** scientificName: *Nyssomyia ylephiletor* (Fairchild & Hertig, 1952); **Location:** country: Panama; stateProvince: Bocas del Toro; locality: Almirante, Finca Nievecita; **Event:** eventDate: 06-22-50; eventRemarks: http://phlebotominaenhmtypes.myspecies.info/node/87; **Record Level:** institutionCode: NHMUK; basisOfRecord: Preserved Specimen
- **Type status:** Paratype. **Occurrence:** catalogNumber: BMNHE1251318; sex: Female; **Taxon:** scientificName: *Nyssomyia ylephiletor* (Fairchild & Hertig, 1952); **Location:** country: Panama; stateProvince: Bocas del Toro; locality: Almirante; **Event:** eventDate: 06-29-51; eventRemarks: http://phlebotominaenhmtypes.myspecies.info/node/90; **Record Level:** institutionCode: NHMUK; basisOfRecord: Preserved Specimen

##### Distribution

Belize, Colombia, Costa Rica, Ecuador, Guatemala, Honduras, Mexico, Nicaragua, Panama

##### Notes

Valid species in *Nyssomyia*.

#### Nyssomyia yuilli

Young & Porter, 1972

Lutzomyia yuilli Young & Porter, 1972 (Young and Porter 1972)

##### Materials

- **Type status:** Paratype. **Occurrence:** catalogNumber: BMNHE1722056; sex: Male; **Taxon:** scientificName: *Nyssomyia yuilli* (Young & Porter, 1972); **Location:** country: Colombia; stateProvince: Antioquia; municipality: Zaragoza; locality: 24km S & 21 km W of Zaragoza; **Event:** eventDate: 23/05/70; eventRemarks: http://phlebotominaenhmtypes.myspecies.info/node/175; **Record Level:** institutionCode: NHMUK; basisOfRecord: Preserved Specimen
- **Type status:** Paratype. **Occurrence:** catalogNumber: BMNHE1722057; sex: Female; **Taxon:** scientificName: *Nyssomyia yuilli* (Young & Porter, 1972); **Location:** country: Colombia; stateProvince: Antioquia; municipality: Zaragoza; locality: 24km S & 21 km W of Zaragoza; **Event:** eventDate: 12/09/70; eventRemarks: http://phlebotominaenhmtypes.myspecies.info/node/176; **Record Level:** institutionCode: NHMUK; basisOfRecord: Preserved Specimen

##### Distribution

Bolivia, Brazil, Colombia, Ecuador, Peru, Venezuela

##### Notes

Valid species in *Nyssomyia*.

## Supplementary Material

XML Treatment for Pressatia acanthobasis

XML Treatment for Psathyromyia aclydifera

XML Treatment for Nyssomyia anduzei

XML Treatment for Pintomyia aulari

XML Treatment for Trichophoromyia auraensis

XML Treatment for Psychodopygus bispinosus

XML Treatment for Lutzomyia botella

XML Treatment for Brumptomyia brumpti

XML Treatment for Micropygomyia cayennensis braci

XML Treatment for Micropygomyia cayennensis hispaniolae

XML Treatment for Micropygomyia cayennensis jamaicensis

XML Treatment for Micropygomyia cayennensis maciasi

XML Treatment for Micropygomyia cayennensis puertoricensis

XML Treatment for Psathyromyia coutinhoi

XML Treatment for Micropygomyia cubensis

XML Treatment for Psathyromyia dasymera

XML Treatment for Dampfomyia del-pozoi

XML Treatment for Micropygomyia duppyorum

XML Treatment for Pressatia dysponeta

XML Treatment for Pintomyia evansi

XML Treatment for Trichophoromyia flochi

XML Treatment for Evandromyia gasti

XML Treatment for Lutzomyia gonzaloi

XML Treatment for Migonemyia hansoni

XML Treatment for Lutzomyia hartmanni

XML Treatment for Evandromyia inpai

XML Treatment for Dampfomyia isovespertilionis

XML Treatment for Psychodopygus lainsoni

XML Treatment for Micropygomyia lewisi

XML Treatment for Warileya lumbrerasi

XML Treatment for Lutzomyia monzonensis

XML Treatment for Pintomyia odax

XML Treatment for Oligodontomyia oligodonta

XML Treatment for Bichromomyia olmeca novica

XML Treatment for Bichromomyia olmeca olmeca

XML Treatment for Pintomyia oresbia

XML Treatment for Lutzomyia peruensis

XML Treatment for Warileya phlebotomanica

XML Treatment for Pintomyia pia

XML Treatment for Evandromyia pinottii

XML Treatment for Pintomyia quasitownsendi

XML Treatment for Nyssomyia richardwardi

XML Treatment for Dampfomyia rosabali

XML Treatment for Trichophoromyia rostrans

XML Treatment for Warileya rotundipennis

XML Treatment for Evandromyia sallesi

XML Treatment for Lutzomyia scorzai

XML Treatment for Nyssomyia shawi

XML Treatment for Pintomyia spinicrassa

XML Treatment for Lutzomyia strictivilla

XML Treatment for Nyssomyia trapidoi

XML Treatment for Micropygomyia trinidadensis

XML Treatment for Trichopygomyia triramula

XML Treatment for Trichophoromyia ubiquitalis

XML Treatment for Nyssomyia umbratilis

XML Treatment for Lutzomyia vexillarius

XML Treatment for Dampfomyia viriosa

XML Treatment for Evandromyia walkeri

XML Treatment for Pintomyia xerophila

XML Treatment for Nyssomyia ylephiletor

XML Treatment for Nyssomyia yuilli

## Figures and Tables

**Figure 2a.**
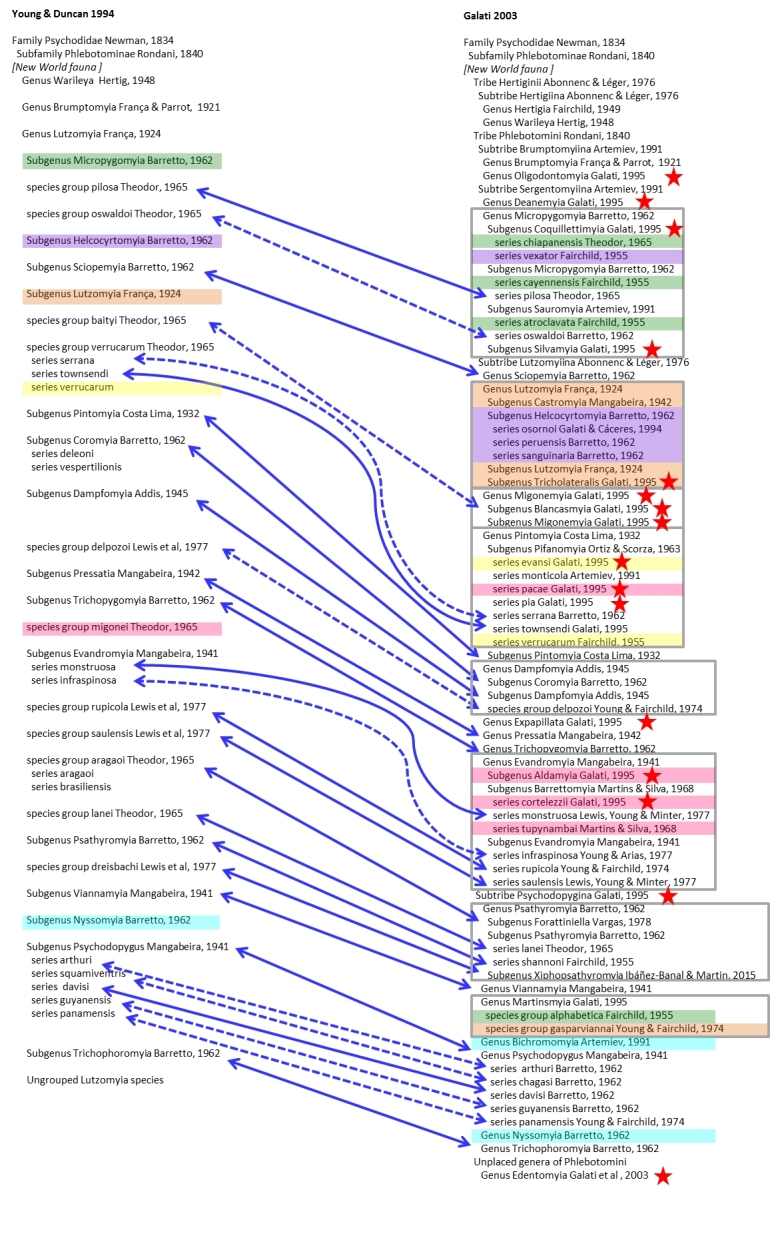
Comparison of the two classifications.

**Figure 2b.**
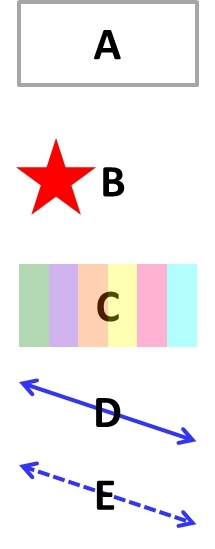
Explanation of the symbols used in Figure 2a. Grey boxes (**A**) on the Galati (2003b) classification indicate novel phylogenetic information provided by the classification of Galati. Aside from a few remarks given in the text, Young and Duncan (1994) give no information on the phylogenetic relationships between any of the sub-generic groups within their genus *Lutzomyia*. Red stars (**B**) indicate new sub-genera or genera in the Galati classification that do not exist in the Young and Duncan classification. Coloured highlighting (**C**) indicates groupings from Young and Duncan which are not recovered as monophyletic groups in the Galati classification. Solid blue arrows (**D**) indicate where exactly the same grouping is recovered in both classifications. Broken blue arrows (**E**) indicate where broadly the same grouping is recovered in both classifications with only very minor changes to the taxa included.
